# Rapid diversification underlying the global dominance of a cosmopolitan phytoplankton

**DOI:** 10.1038/s41396-023-01365-5

**Published:** 2023-02-06

**Authors:** El Mahdi Bendif, Ian Probert, Odysseas A. Archontikis, Jeremy R. Young, Luc Beaufort, Rosalind E. Rickaby, Dmitry Filatov

**Affiliations:** 1 Department of Earth Sciences, University of Oxford, Oxford, UK; 2 Department of Plant Sciences, University of Oxford, Oxford, UK; 3 Institut des sciences de la mer de Rimouski (ISMER), Université du Québec à Rimouski, Rimouski, Canada; 4 Sorbonne Université – CNRS, Roscoff Culture Collection, FR2424 Station Biologique de Roscoff, Roscoff, France; 5 Department of Earth Sciences, The Natural History Museum, London, UK; 6 Department of Earth Sciences, University College London, London, UK; 7 Aix Marseille Université, CNRS, IRD, INRAE, CEREGE, Aix-en-Provence, France

**Keywords:** Phylogenomics, Population genetics, Microbial biooceanography

## Abstract

Marine phytoplankton play important roles in the global ecosystem, with a limited number of cosmopolitan keystone species driving their biomass. Recent studies have revealed that many of these phytoplankton are complexes composed of sibling species, but little is known about the evolutionary processes underlying their formation. *Gephyrocapsa huxleyi*, a widely distributed and abundant unicellular marine planktonic algae, produces calcified scales (coccoliths), thereby significantly affects global biogeochemical cycles via sequestration of inorganic carbon. This species is composed of morphotypes defined by differing degrees of coccolith calcification, the evolutionary ecology of which remains unclear. Here, we report an integrated morphological, ecological and genomic survey across globally distributed *G. huxleyi* strains to reconstruct evolutionary relationships between morphotypes in relation to their habitats. While *G. huxleyi* has been considered a single cosmopolitan species, our analyses demonstrate that it has evolved to comprise at least three distinct species, which led us to formally revise the taxonomy of the *G. huxleyi* complex. Moreover, the first speciation event occurred before the onset of the last interglacial period (~140 ka), while the second followed during this interglacial. Then, further rapid diversifications occurred during the most recent ice-sheet expansion of the last glacial period and established morphotypes as dominant populations across environmental clines. These results suggest that glacial-cycle dynamics contributed to the isolation of ocean basins and the segregations of oceans fronts as extrinsic drivers of micro-evolutionary radiations in extant marine phytoplankton.

## Introduction

Marine phytoplankton contribute to about half of global primary productivity, and play a key role in the ocean ecology and the climate system [1]. Planktonic populations are generally characterised by very high species diversity, but only a limited number of species dominate productivity and biomass [2]. These cosmopolitan “keystone” species, traditionally defined according to morphological characters, are increasingly being shown to comprise more than one genetic entity [3–6], hence forming complexes of “cryptic” or “pseudocryptic” species [7]. Nevertheless, very little is known about the micro-evolutionary processes underpinning cryptic and pseudo-cryptic speciation [8]. Divergent selection usually leads to the formation of species with distinctive morphological features, it can sometimes affect traits related to reproductive isolation without triggering morphological change [9]. For example, reproductive isolation resulting from the fixation of incompatibilities in separate populations subject to similar selective pressures predisposes the origination of cryptic species [10]. Cosmopolitan phytoplankton, which form highly dispersed populations experiencing a range of selective pressures, many of which likely overlap [11], thus represent highly pertinent models for the study of evolutionary processes leading to cryptic speciation. This is especially relevant for planktonic species bearing hard shells or skeletons harbouring diverse structural formations, classically used as relevant diagnostic characters for their taxonomy. Their fossil record attests to the emergence and extinction of such predominant species and provides useful zonation for biostratigraphers [12]. On geological timescales, these dominances or “acmes” have been found sometimes associated to macroevolutionary processes, such as size variation through time [13–15], potentially corresponding to the successive dominance of distinct species with similar morphologies [16–18].

Coccolithophores (Calcihaptophycidae, Haptophyta), a key group of marine phytoplankton, produce microscopic calcite platelets, called coccoliths, that cover the cell [19]. Since their origin in the Triassic, they have left a significant fossil record, attesting to their importance in biogeochemical cycles [20]. The production of coccoliths at the sea surface and their subsequent sinking to depth impact upper-ocean alkalinity, which has a significant influence on the exchange of CO_2_ between the sea and the atmosphere [20]. Coccolithophores have been characterised according to a morphological species concept (i.e., differences in shape and arrangement of crystal units forming coccoliths), used to distinguish “morphospecies” [21]. In addition, relatively minor differences (i.e., size of crystals) have also been used to differentiate “morphotypes” which are generally inferred to be intra-specific variants [21, 22]. However, the significance of these morphological subtleties remains to be assessed according to a biological species concept [23]. If morphotype differences align with genomic variability, the study of evolutionary genetics can be integrated with that of the fossil record to shed light on mechanisms of diversification in phytoplankton [17, 24, 25].

Of the ca. 250 described extant species, *Gephyrocapsa huxleyi* [17, 19] (commonly known as *Emiliania huxleyi* [26]) is the most abundant and widespread coccolithophore. Defined as one of the most successful marine phytoplankton, it is thought to be also one of the main calcite producers on Earth [20]. Present in almost all oceans, *G. huxleyi* populations regularly form extensive “white water” blooms in high latitude coastal and shelf ecosystems that contribute significantly to the biological carbon pump [20, 27]. Moreover, *G. huxleyi* belongs to the *Gephyrocapsa* genus which dominates the fossil record of the last 2 Ma, while demonstrating a cyclical pattern of successive coccolith size changes [17]. The most recent of these cycles corresponds to the radiation of at least six extant morphospecies that includes *G. huxleyi* [17, 25]. Although this morphospecies first appeared around 290 ka [12], it now shows significant morphological diversity, with a range of described morphotypes that are considered to divide into two main morphogroups, A and B [28–33] (Supplementary Fig. S1, Supplementary Table S1). Observations of natural assemblages have revealed that different morphotypes dominate in distinct oceanic regions [31, 32, 34, 35], perhaps being selected by seasonal fluctuations of environmental factors [36]. In addition, cultured strains of different morphotypes have been shown both to retain their distinctive morphological features over time and to display contrasting responses to changes of temperature [37] and pH in the growth medium [38, 39], two abiotic predicators known to selectively influence phytoplankton communities [40, 41]. These empirical observations suggest that morphotypes may have evolved as distinct genotypes through ecological selection in diverse habitats [42]. Moreover, morphotypes may differentially influence carbon fluxes since they can vary significantly in the degree of their calcification [20, 43].

Phylogenetic studies on *Gephyrocapsa* based on single genes have produced inconsistent results and only limited congruence with morpho-taxonomy [44], mostly due to a lack of resolution. However, recent genomic surveys have established a concordance between extant morphospecies and biological species and revealed patterns of diversification between *Gephyrocapsa* species [25, 45, 46]. These investigations reconciled macro-evolutionary patterns observed in the fossil record with genetic processes underlying speciation in marine phytoplankton alongside glacial cycles [17, 25]. A more detailed genomic comparison of *G. huxleyi* strains could resolve the genetic delineation of morphotypes, while providing insights into intra-specific patterns of diversification and adaptation in marine phytoplankton.

In this study, we leveraged the reference *G. huxleyi* genome sequence and availability of numerous strains in culture to address whether morphotypes coincide with genetic variability in *G. huxleyi*. We also assessed how *G. huxleyi* populations diversified into distinct morphotypes through recent climatic oscillations. This study built on previous investigations of *Gephyrocapsa* diversity [17, 25, 45–47], adding newly sequenced genomes from a collection of morphologically defined clonal cultures originating from worldwide locations (Supplementary Fig. S2; Supplementary Tables S2-3). This provided genome-wide sequence data of 59 isolates (29 newly genome-wide sequence data in addition to 30 already published) that we aligned against the reference (CCMP1516) *G. huxleyi* genome [46] in order to reconstruct the evolutionary ecology of this keystone lineage since its appearance.

## Materials and methods

### Origin and morphological characterisation of analysed strains

Clonal *Gephyrocapsa* strains (Supplementary Table S1) from the Roscoff Culture Collection (RCC; roscoff-culture-collection.org) were maintained in K/2 (-Si,-Tris,-Cu) medium at 17 °C with 50 µmol-photons.m^-2^.s^-1^ illumination provided by daylight neon tubes with a 14:10 h L:D cycle. Samples were collected during late exponential phase before filtration using a 0.45 µm cellulose nitrate membrane filter, which were then mounted onto metallic stubs using adhesive tape and gold-coated using a sputter coater. Coccoliths and coccospheres were visualised using a Phenom ProX Desktop SEM (Phenom-World, Eindhoven, Netherlands) at the Station Biologique de Roscoff, France, on a Phillips XL-30 FEG field emission SEM (FEI, Eindhoven, Netherlands) and an Ultra Plus Zeiss at the facilities of the Natural History Museum, London, UK. Scanning electron micrographs were captured at magnifications ranging between ×8000 and ×20,000, and electron beam damage was minimised by operating the microscope at 15 kV. Morphometric measurements were carried out on the length of coccoliths, being the usual character measured for estimates of carbonate flux [48], with a minimum of 60 isolated coccoliths analysed per sample. See Supplementary Information for further details.

### DNA extraction

Cells of 29 strain cultures (additional to previous dataset [25]) were harvested by centrifugation at 4500 g for 15 min. They were then washed twice with TE buffer and suspended in 10 ml of lysis buffer (Tris, 0.1 M; EDTA, 0.05 M; NaCl, 0.1 M; 1% SDS; 2% N-lauroylsarcosine, proteinase K 200 mg/mL, pH 8.0) before incubation at 55 °C for 2 h. DNA was then purified with equal volumes of phenol and chloroform and precipitated with ethanol. For each sample, quantifications and quality of nucleic acids were performed with a Qubit 3.0 fluorometer (Thermofisher Scientific, Inc.) and a Nanodrop. DNA extracts were then sent to the Wellcome Trust Centre for Human Genomics, Oxford (WTCHG) for sequencing. Paired-end libraries were prepared individually, barcoded, and then combined prior to sequencing. Libraries were sequenced using a HiSeq 2500 (Illumina) sequencing platform to produce 150 base-pair (bp) paired-end reads. The amount of raw data generated for each strain is listed in Supplementary Table S2.

### Mapping of reads

After quality trimming with Trimmomatic [49], the sequence reads from 59 *Gephyrocapsa* strains were mapped to the *G. huxleyi* CCMP1516 reference genome (N50: 408.69 kb; median length: 1.77 kb; average length: 26.25 kb) [46] with BWA-MEM [50] as in [17]. Despite low sequence divergence (<3% total sites) between the strains analysed, the proportion of reads mapped to reference was relatively low (25–69%; Supplementary Table S2) more likely due to the lack of coverage in some sequenced strains with a large proportion of the genome missing than the potential variability of *G. huxleyi* pan-genome (Mapping coverage <45% with a breadth of coverage <20×; Supplementary Table S2). For most analyses in this study, 47 strains with the best mapping coverage (>20×) were retained for further filtering prior to downstream analyses. See Supplementary Information for further details.

### Population structure analysis

In order to evaluate the genetic structure of *G. huxleyi*, 47 vcf files with greatest coverage were merged using the BCFtools program [51]. The obtained vcf file was then filtered to remove indels. Only biallelic variants with a minor allele frequency above 0.05 to avoid duplicates, and with mean depth of coverage between 2 and 50 were retained. This resulted in a total of 2,086,643 biallelic single nucleotide polymorphisms (SNPs). The vcf file was converted as a genlight object before performing analyses through the *adegenet* package [52] in R. A first PCA was performed with the *glpca* command for a first assessment of genetic clusters. For comparison, we conducted a k-means research (*find.clusters*) in order to implement a DAPC. All *adegenet* analyses were then visualised using *ggplot*. Pairwise differentiation index (*F*_ST_) [53] were calculated per site between pairs of lineages using the function s*stamppFst*, through the *stampp* package [54].

### Phylogenetic inferences

Phylogenetic tree reconstructions were conducted for two datasets, one comprising 47 strains and another with 59 strains. For both datasets, a multi-species coalescent-based and a concatenated approach were performed. See Supplementary Information for further details.

For a visual comparison of clades and sub-clades in relation to the D-suite analysis we generated a neighbour-net splits network [55] in SplitsTree4 [56]. This analysis was conducted on a SNP alignment deduced from the 47 best-covered genomes by using vcftools to remove SNP sites with less than 25% missing data, which reduced the full dataset from 2,086,643 to 85,365 biallelic SNPs.

### Species delimitation

We assessed the fit of alternative scenarios of species delimitation in *G. huxleyi* under a coalescent framework. Bayes Factor Delimitation (BFD) [57] were used as this method was implemented for genome-wide SNP data in the package snapper v1.0.2 [58] within the BEAST2 v2.6.3 [59] program. For all models, we conducted the analysis on our dataset containing 85,365 biallelic SNPs using a path sampling of 18 steps (1,000,000 Monte Carlo Markov chain (MCMC) iterations, with a 20% burn-in), with a log-likelihood correction. A gamma distribution was implemented on the tree height, lambda parameter, as Maximum Likelihood Estimations (MLE) are affected by improper prior distributions. Bayes factors (BF) were calculated from the MLE for each model following this formula [57]:

$${{{{{{{\mathrm{BF}}}}}}}} = 2\,{{{{{{{\mathrm{x}}}}}}}}\,({{{{{{{\mathrm{MLE}}}}}}}}_{{{{{{{{\mathrm{null}}}}}}}}}-{{{{{{{\mathrm{MLE}}}}}}}}_{{{{{{{{\mathrm{test}}}}}}}}})$$

Positive BF values indicate support for the null model and negative values favour the tested model.

### Gene flow analysis

Further assessment of gene flow between *G. huxleyi* entities were conducted with Dsuite [60] by estimating *D*-statistics for all possible trios representing the different populations. See Supplementary Information for further details.

### Divergence times

In order to evaluate divergence times within *G. huxleyi*, we used BEAST v2.6.3 [59] using 50 randomly selected from 5 kb supergene alignments composed of 59 strains of *G. huxleyi*. See Supplementary Information for further details.

### Demographic modelling

To explore alternative demographic models, we used the diffusion approximation method of dadi [61] to analyse joint site frequency spectra. 20 demographic models were fit, for which coalescent parameters were inferred using the dadi_pipeline v3.1.5 [62] (https://github.com/dportik/dadi_pipeline). The scenario obtaining the highest likelihood and the best information criterion (AIC) was deemed the most probable model. See Supplementary Information for further details.

### Environmental parameters

Average sea surface temperature, nutrients (nitrate and phosphate), and carbonate chemistry at the strain isolation sites (resolution of 5° squares) and at the month and year of collection were determined using data from World Ocean Database 2018 [63] and modelled data OceanSODA-ETHZ [64] both available from the National Oceanic and Atmospheric Administration (https://www.ncei.noaa.gov/. Accessed 01/02/2022). See Supplementary Information for further details.

## Results and discussion

### Genetic and morphological delineation between G. huxleyi strains

**Fig. 1:**
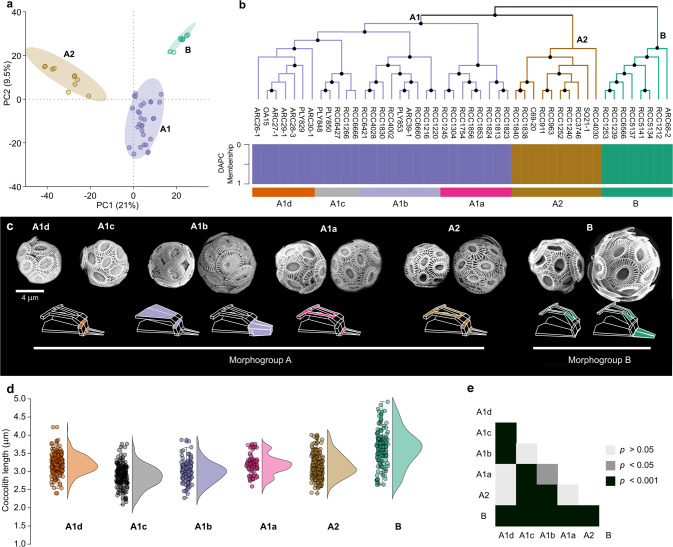
Relationship between genetic structure and morphotypes in *G. huxleyi*. **a** Principal component analysis (PCA) based on 2,086,643 SNPs recovered from 47 *G. huxleyi* genomes; **b** Relationship between coalescent species phylogeny (ASTRAL tree based on 1000 supergenes) and DAPC clustering; **c** Correspondence between morphotypes and lineages within *G. huxleyi*, and sub-lineages within A1 (scale bar = 4 μm). Variable elements in relation to genotypes are highlighted in the schematics under the SEM pictures; **d** Distribution of coccolith length for 5 randomly chosen strains representing each clade and sub-clade, with a jittered box-plot on the left and a half-violin plot on the right for each group; **e** Matrix plot of Bonferroni corrected *p*-value corresponding to the Dunn-test for the comparison of coccolith length measurements between groups.

We first assessed genetic variability through analysis of genomic polymorphism to determine whether distinct genetic lineages exist in *G. huxleyi* and to test whether these relate to morphotypes. We used 2,086,643 high-quality biallelic single nucleotide polymorphisms (SNPs) retrieved from the 47 clonal culture strains with the best genome sequence coverage (>20×). A principal component analysis (PCA) and a discriminant analysis in principal component (DAPC) both delineate three well-defined genetic groups, with the distribution of strains being unequal and with no overlap on the principal components (Fig. 1a; Supplementary Fig. S3a,b). With regards to population structure, the DAPC analysis suggested that 3 clusters (K = 3) can be used to depict a genotype membership matrix for each strain (Fig. 1b; Supplementary Fig. S4). As such, it confirmed the three-lineage delineation proposed by the PCA, while illustrating no admixture between lineages.

Phylogenetic inference based on alignments with higher mapping coverage only (47 strains) or including sequences with lower mapping coverage (59 strains) all supported segregation of strains into three main lineages, which we term clades A1, A2 and B, with A1 and A2 being more closely related to each other than to B (Fig. 1b; Supplementary Fig. S5a, b). This delineation is congruent with previous studies on the phylogeny of the *Gephyrocapsa* genus [17, 46, 65]. These clades also correspond to differences in morphotypes (Fig. 1b, c). All strains in clade A1 produce unambiguous A-group coccolith morphotypes (type A and type R). Similarly, all strains in clade B produce unambiguous B-group coccolith morphotypes (type B and type O). Clade A2 is less distinctive, with strains producing lightly calcified type A coccoliths. Some of these strains could be classified as type B/C [66] or C (both regarded as B-group morphotypes), but distinctive by the lower elevation of distal shield elements and by greater degree of calcification of the central area grid (which is reduced and sometimes absent in morphotypes B/C and C). At a finer level, clade A1 is composed of four sub-clades, which we term A1a, A1b, A1c, and A1d. Strains in sub-clades A1a, A1c and A1d all produce coccoliths with type A morphologies and distinctive degrees of calcification: strains in the sub-clade A1a form relatively lightly calcified coccoliths with regular elements, while strains in sub-clades A1c and A1d produce similar moderately calcified coccoliths, sometimes with conspicuous irregularities (inner tube elements overlapping into the central area). Strains in clade A1b produce distinct coccoliths exhibiting A-group morphology but with heavy calcification, including forms with heavily calcified shields which have been termed type R and also forms with heavily calcified central areas which have been referred to as “type A overcalcified”. Some clade A2 strains produce coccoliths with a similar morphology to strains in A1a, indicative of partially cryptic lineages (Supplementary Fig. S2; Supplementary Table S4).

The congruence between morphotypes and clades is also supported by significant differences in the length of coccoliths measured between some of the clades (Fig. 1d, e). The morphogroups A and B differ significantly, and insignificant comparison relates to the comparison of sub-clades against the clade A2, which reinforces the closest relationship between A1 and A2. We denote also that the case of A1a and A2 demonstrating no significant difference in coccolith length concurs with the cryptic delineation mentioned above.

Based on the clustering analyses and the phylogenetic reconstructions, we tested whether different groupings are distinct species with regards to the null hypothesis “*G. huxleyi* is a single species”, which correspond to the current state of taxonomy. Species delimitation based on comparison of Marginal Likelihood Estimators (MLE) with Bayes Factors (BF) supported the hypothesis that the three lineages depicted by ordination and phylogenetic reconstructions are distinct species as the best model (Table 1).

**Table 1:** Species delimitation based on Bayes Factor Delimitation (BDF).

Hypothesis	Number of species	MLE	BF	Rank
*Gephyocapsa huxleyi* is a single species (Null hypothesis)	1	−53,986	0	4
*Gephyrocapsa huxleyi* is composed of two morphospecies (morphogroups A and B)	2	−54,078	184	5
*Gephyrocapsa huxleyi* is composed of three species, as suggested by the PCA (lineages A1, A2 and B)	3	**−53,558**	−**856**	**1**
*Gephyrocapsa huxleyi* is composed of five species (two within the A morphogroup and three within the B morphogroup)	5	−53,569	−834	2
Each sub-clade (e.g., A1a, A1b, A1c, A1d) is a distinct species	8	−53,708	−556	3

The best model is shown in bold.

*MLE* Maximum Likelihood Estimation, *BF* Bayes Factor.

**Fig. 2:**
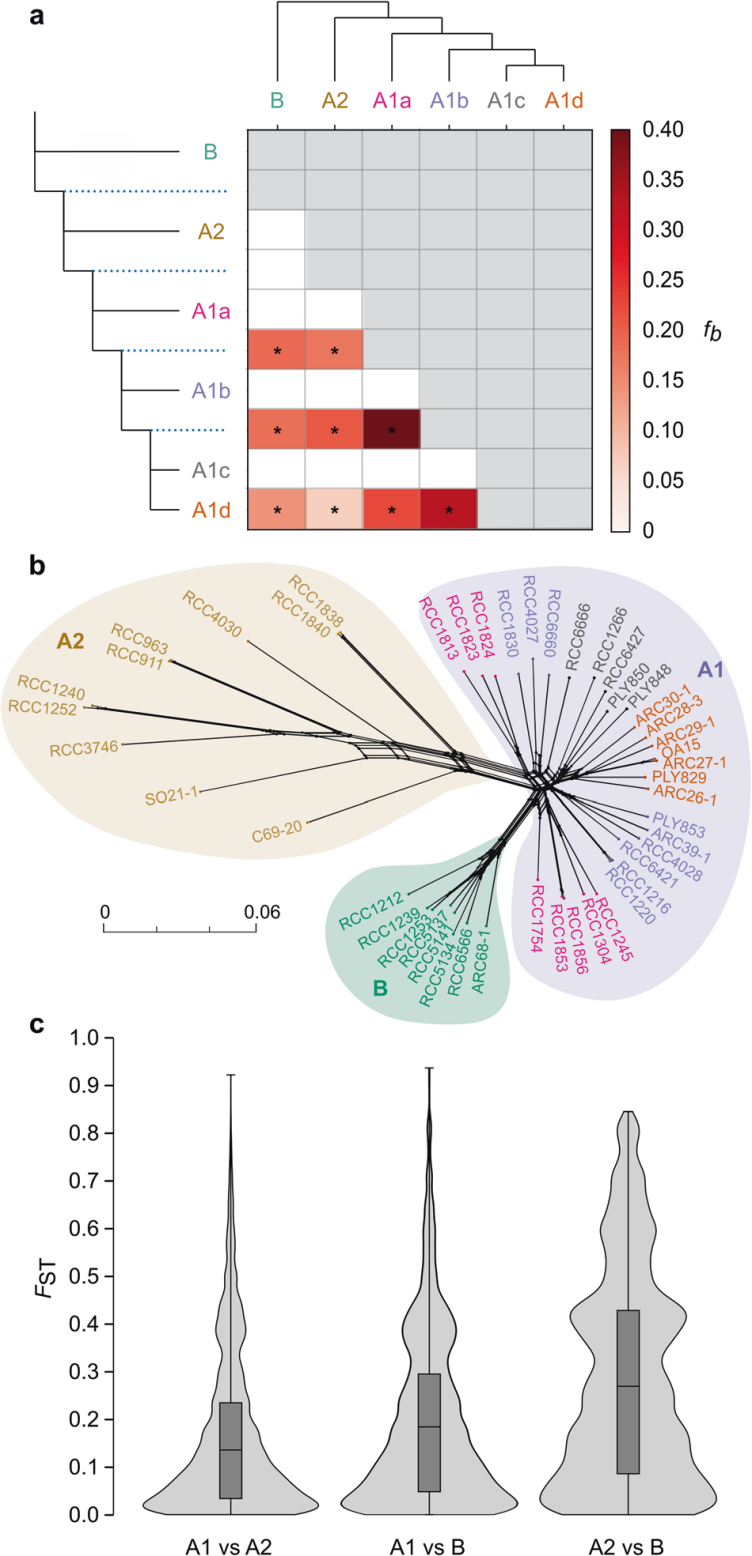
Excess of allele sharing and differentiation in *G. huxleyi*. **a** *f-branch* (*f*_*b*_) statistics between lineages and sub-lineages. The gradient represents the *f*_*b*_ score, grey blocks represents tests not consistent with the species tree (for each branch on the topology of the y axis, having itself or a sister taxon as donor on the topology of the x axis); asterisks denote block jack-knifing significance at *p* < 0.05 (after Bonferroni correction); **b** Phylogenetic network inferred using a subset of 83,563 SNPs across 47 strains; **c** Combined box and violin plots showing the distribution of genetic differentiation per sites between lineages for synonymous sites.

D-statistics calculated to estimate gene flow reveal a non-significant excess of alleles shared between the three lineages (Fig. 2a; Supplementary Table S5). *Fbranch* statistics, (*f*_*b*_) revealed significant signatures of gene-flow between sub-lineages within A1 associated with correlated estimates in relation to A1a, A2 and B (Fig. 2a) [60]. Signatures on the basal branch of diversification in A1 may correspond to genetic exchanges between A1 and B, with gene-flow signatures attributed to A2 corresponding to correlated estimates due to common ancestry. Recent signatures of gene-flow throughout the evolution of A1 are thus likely associated to the common ancestry between A1a, A2 and B during gene-flow events between the sub-lineages, as supported by the non-significant *D* statistics between the three lineages. Moreover, the phylogenetic network revealed similar convolutions between A1 sub-lineages but clear separation of the main lineages and longer branches in the A2 lineage (Fig. 2b).

Comparison of pairwise differentiation estimates per sites (*F*_ST_), for synonymous sites, supported A1 and A2 to be more closely related (*F*_ST_ = 0.153) than A1 and B (*F*_ST_ = 0.191) as depicted by the phylogenies (trees and network). Although, the differentiation between A2 and B was more marked than any other comparison (*F*_ST_ = 0.276; Fig. 2c; Supplementary Tables S6-7) which could relate to the long branches observed in the phylogenetic network (Fig. 2b). These results rather suggest a rapid divergence within the clade A2, likely since its emergence.

These analyses of genetic divergence and phylogenetic relationship therefore indicate that *G. huxleyi*, usually considered a single species, has differentiated into at least three reproductively isolated species, A1, A2 and B. These results expand on recent phylogenomic and population genetic results [17, 25], and support the binary morphogroup classification [28]. Within these lineages, morphotypes are structured into distinct clades, providing clear evidence that *G. huxleyi* morphotypes correspond to distinct genotypes.

### Environmental drivers of diversification in the three species of Gephyrocapsa

**Fig. 3:**
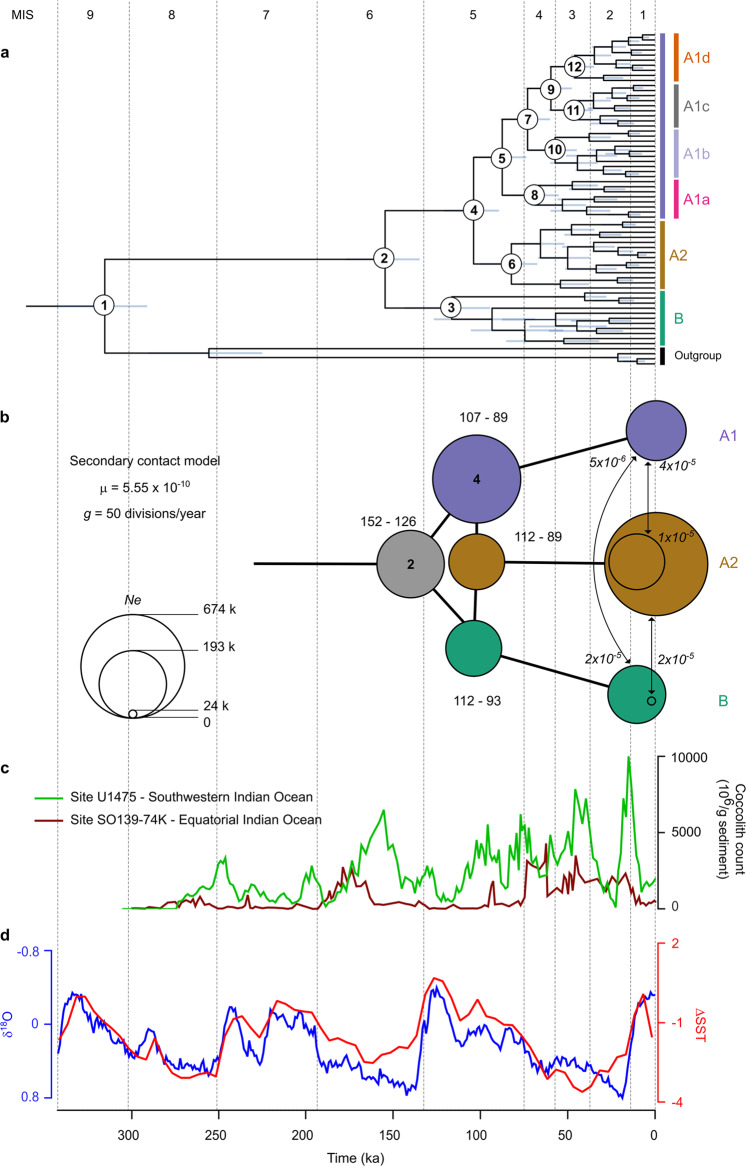
Tempo of diversification between and within lineages in *G. huxleyi*. **a** Phylogenetic chronogram of *G. huxleyi* based on analysis of genome sequence data of 63 strains. The phylogeny was rooted using one strain of *G. muellerae* and three strains of *G. ericsonii/parvula* as an outgroup. Every node in the phylogeny has a posterior probability at 1. The dating of ancestral nodes is based on relaxed molecular clock calibrated with the first appearance of *G. huxleyi* (node 1; 290 ka) in the fossil record. Details for nodes are reported in Supplementary Table S7. 95% Highest posterior density intervals for ages are shown as grey bars. **b** Visual representation of the parameters inferred for consecutive speciation events between lineages A1, A2 and B. Circles reflects effective population size (*Ne*) estimated for extant and ancestral species, on nodes and on leaves. Node 2 and 4 are highlighted in bold for correspondence with the chronogram. Divergence time are provided as an interval accounting for *μ* (=mutation rate) uncertainty [86]. Arrows represent secondary contact events with migration values in italic. All parameter estimates are listed in Supplementary Table 9. *g*: generation time. **c** Absolute abundance of *G. huxleyi* in sites U1475 [87] and SO139-74KL [88]. **d** Global Δ Sea Surface Temperature [89] (ΔSST; blue line) and LR04 [90] (red line) over 350 ka; (MIS: Marine Isotopic Stage).

We established a timeline of diversification by comparing the fossil record attributed to *G. huxleyi* with genomic divergence time based on a molecular clock reconstruction and joint-site frequency spectrum (JSFS) modelling (Fig. 3; Supplementary Table S7, Supplementary Fig. S5). According to sedimentary records, first occurrences (FO) of *G. huxleyi*-like ancestors occurred synchronously across low latitude sites during glacial stage MIS8 around 290 ka [12]. We used this date to calibrate our chronogram (Fig. 3a; Supplementary Table S7).

**Fig. 4:**
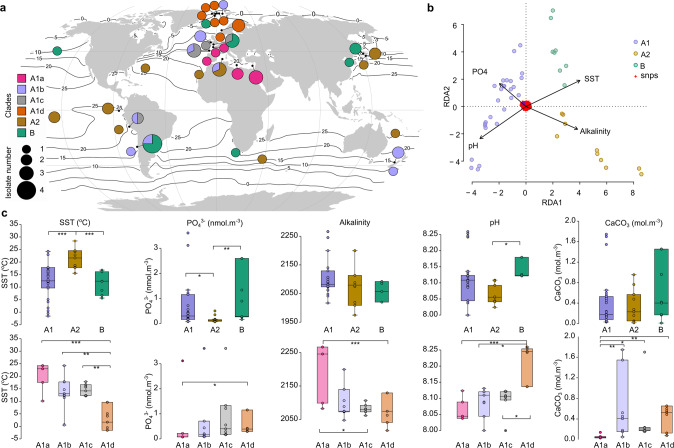
Relationship between genetic lineages and environmental variables. **a** Distribution map of lineages and sub-lineages based on strains used in this study. **b** Redundancy Analysis plot with constrained predicators. **c** Strain distributions lineages/ and sub-lineages per relevant environmental parameters (significances (*p* values) of the Dunn test correspond to: *p < 0.05; **p < 0.01, ***p < 0.001).

Our divergence time reconstruction indicates that modern *G. huxleyi* populations originated from the divergence of A and B clades in the MIS6 glacial period preceding the end of the Pleistocene (152 (124–185) ka in our study and ~170 (88–223) ka in [17]), which corroborates the time range deduced from sedimentary observations for the appearance of B-like morphotypes [67, 68]. Our JSFS analysis supported this divergence, but as an episode of geographic separation (vicariance) and niche partitioning between A1 and B (138 (126–152) ka; Fig. 3b). Sharper isolation of fronts and stronger physical structuring during this glacial period could have accounted for this divergence in isolating distinct populations. In contemporary oceans, the partially cryptic A1a and A2 clades have sympatric distributions associated with low latitude and warm water masses. Sub-clades A1c and A1d together with clade B form another sympatric assemblage, but associated with temperate and subpolar waters. There is limited overlap between the distributions of these two groupings, which therefore form allopatric assemblages defined by their ecologies. Although, the distribution of strains belonging to sub-clade A1b does overlap with those of A1a, A2 and B (Fig. 4a). Accordingly, and given the basal divergence of A1a in A1, it is likely that A1, A2 and B lineages have been diverging with distinct preferences to sea surface temperature, nutrients concentrations and carbonate chemistry, as suggested by the redundancy analysis (RDA; Fig. 4b, c).

Further diversifications followed during the MIS5 interglacial period, with a significant discrepancy between the chronogram and JSFS-modelled divergence. While the chronogram suggests diversification occurred within B (115 (78–170) ka and 92 (68–125) ka) and between A1 and A2 (103 (88–119) ka), the genetic model indicates that A1 and A2 diverged around the same time as A2 and B (respectively 102 (93–112) and 98 (89–107) ka; Fig. 3b; Supplementary Table S7). This result reinforces the view that the earliest divergence was between A1 and B and indicates a convoluted divergence for A2 (cf. fbranch and phylogenetic network; Fig. 2a, b), which could be the result of hybridisation during early stage of speciation. It confirms gene flow signatures associated with potential interaction between A1 and B prior to diversification within a branch of A1 (Fig. 2a). The three divergences within *G. huxleyi* tested with JSFS retrieved a similar mode of speciation as for the more general *Gephyrocapsa* group, that has undergone speciation events followed by occasional gene flow during secondary contacts [25]. In *G. huxleyi*, putative secondary contacts occurred recently during MIS1 with an extremely reduced measure of gene-flow (M < 1; 10^−6^ < M < 10^−5^; M: Migration, number of individuals from a population that exchanged gene with another), confirming non-significant *D-statistics* found between the lineages. Overall, these major divergence events were not restricted to a particular environmental scenario, but rather to the fluctuating state of MIS5 in terms of niche expansion and compression.

The first divergences within B and A2 are associated with sub-clades composed of strains originating from distinct oceans, suggesting patterns of geographic isolation, as a result of migrating fronts in relation with gradual ice growth that followed the interglacial maximum. Distinct morphotypes are associated with geographic separations within B, morphotype B being found in the Atlantic Ocean and morphotype O in the Pacific Ocean, while morphological variations are less consistent in A2 (Supplementary Figs. S1–2 and Supplementary Tables S1-2 and S4). Within A1, intra-specific divergences then occurred through different events of vicariance during the MIS4-2 glacial period, establishing genetically distinct populations along a latitudinal gradient. A significant eccentricity minimum associated with these events could account for interactions between newly diversified populations within A1 due to stronger compression of ecological niches [69]. In this scheme, gene-flow between emerging populations could have played a role in the adaptive process associated with expanded habitat to higher latitudes. Gene flow events within A1 redistributed allelic composition associated with common ancestors of the three lineages into newly formed populations, contributing to potential adaptation to environmental variability along latitudinal gradients. The 400 ky cycle of the absolute eccentricity minimum, which had not occurred since the origin of the *G. huxleyi* lineage, may be related to major events of diversification within *Gephyrocapsa* [69].

Early diversifications within A1 fit with the timing of the diachronic acme (≥50% dominance in the total fossil coccolithophore flora) along a latitudinal gradient (85 ka in low latitudes, 73 ka in transitional latitudes, 61 ka in high latitudes of the North Atlantic Ocean) [70, 71], and were also associated with a notable increase in coccolith size (to >4 µm) until the last glacial maximum (LGM) [72–74]. Physiological experiments have demonstrated that *G. huxleyi* is extremely competitive in certain nutrient limitation scenarios [75, 76]. Therefore, *G. huxleyi* might have benefitted from further fertilisation of the ocean linked to reduced sea level during this glacial period [77], which also contributed to increase seawater alkalinity levels, favouring calcification [78] (as may be now the case in the Black Sea [79], for example). Such bloom-forming conditions would account for modulation of the life cycle toward clonal reproduction [28], leading to increased genetic diversity through fixation of heterozygous substitutions [80]. In this context, distinct populations, perhaps even strains/individuals, may reach reproductive isolation faster, in association with increased probabilities for incompatibilities in offspring during secondary contact [47, 81]. This micro-evolutionary pattern integrates well with previously described patterns of speciation [17, 25], in which macro-evolutionary size variations observed in the fossil record were caused by repeated species radiations rather than fluctuations in the relative abundance of large- and small-celled species. By contrast, interglacials, and especially the current MIS1, may witness the selective impacts of an increase in atmospheric CO_2_ [74] (i.e., global warming), as attested by the gradual reduction of coccolith size and abundance in the fossil record [72–74].

### Taxonomic implications

Based on the results presented herein, we propose a formal taxonomic reassessment that integrates morphological, phylogenetic, admixture, and ecological information relative to the genus as diagnostic features. The species *G. huxleyi* (=clade A1) originated relatively recently along with two other species, leading us to split the entity *G. huxleyi* into three species by emending the former *G. huxleyi*, erecting *G. pseudohuxleyi* sp. nov. (=clade A2), and reinstating *G. pujosae* comb & stat. nov. (=clade B). We believe that this new nomenclature will be useful for future studies of assemblages using (meta)genomic comparison, coupled or not with electron microscopy, taking into account that some populations are (pseudo)cryptic and others may not calcify. This proposal reflects current knowledge of the process of speciation and may change with future evolution of concepts, in the same manner as taxonomic considerations for this complex have evolved over the 20^th^ century [82]. For practical reasons, particularly for studies that do not employ genomic or electron microscopy analyses (as is currently the case for example for most micropalaeontological investigations), these three species can be accommodated into a “superspecies” concept under the name *G. huxleyi*, as has implicitly been the case for years under the name *Emiliania huxleyi*.

#### *Gephyrocapsa huxleyi* (Lohmann) Reinhardt emend. Bendif, Probert, Beaufort, Rickaby & Archontikis

##### Description

Coccoliths with moderately elevated distal shield (2–4 μm length) and elements of variable width (0.05–0.25 μm); inner tube with variable width, sometimes irregular, sometimes irregularly extended on the central area; central area sometimes with a grill of curved rods, sometimes thick lath-lick element forming a solid plate with irregular holes, sometimes strainer-like grill with regular holes, sometimes closed. Comprise previously described morphotypes A, over-calcified and R.

##### Genetic diagnosis

genetically distant from other species of *Gephyrocapsa* by genome sequences. Admixture pattern distinct from *G. pujosae* comb. nov. and *G. pseudohuxleyi* sp. nov. and forms the phylogenetic clade A1 (Fig. 1).

##### Basionym

*Pontosphaera huxleyi* Lohmann 1902 p. 130, pl. 4 Figs. 1–6, pl. 6 Fig. 69 [19].

##### Synonyms

*Hymenomonas huxleyi* (Lohmann) Kamptner [83]; *Coccolithus huxleyi* (Lohmann) Kamptner [84]; *Emiliania huxleyi* (Lohmann) Hay & Mohler [26]; *E. huxleyi* var *huxleyi* Medlin & Green [30].

##### Lectotype

Lohmann 1902 p. 130, pl. 4 Figs. 1–6, pl. 6 Fig. 69 [19].

##### Holotype

Type specimen represented by metabolically inactivated strain RCC1853 cryopreserved at the Roscoff Culture Collection (RCC; roscoff-culture-collection.org). Note: corresponds to isolate collected in the Ionian Sea, which is the type habitat of the original observation.

##### Habitat

Present in all oceans, in water with monthly sea surface temperature ranging from 0 to 25 °C.

#### *Gephyrocapsa pseudohuxleyi* sp. nov Bendif, Probert, Beaufort, Rickaby & Archontikis

##### Description

Coccoliths with moderately elevated distal shield (2–4.5 μm length) and elements of variable width (0.05–0.15 μm); inner tube with variable width, sometimes irregular, central area sometimes with a grill of curved rods, sometimes thick lath-like solid plates (sometimes with irregular holes). Regarded as a variant of morphotype A.

##### Genetic diagnosis

genetically distant from other species of *Gephyrocapsa* by genome sequences. Admixture pattern distinct from *G. pujosae* comb. nov. and *G. huxleyi* and forms the phylogenetic clade A2 (Fig. 1).

##### Holotype

Metabolically inactivated strain PLYM217 cryopreserved at the RCC as RCC1731.

##### Etymology

Based on the contraction of “pseudo-“ and *huxleyi* to highlight the partially cryptic relationship with the former species at the coccolith morphology level.

##### Habitat

Present in all oceans at low latitudes in water with monthly temperature ranging from 15 to 30 °C; can be found in temperate water during summer.

#### *Gephyrocapsa pujosae* Verbeek comb. & stat. nov. emend. Bendif, Probert, Young, Beaufort, Rickaby & Archontikis

##### Description

Coccoliths with distal shield elevated (2.5–5 μm length), composed of wide or narrow shield elements (0.05–0.12 μm width). Central area with thin lath like elements forming sometimes a thin solid plate, sometimes absent. Comprise previously described morphotypes B, B/C, C and O.

##### Genetic diagnosis

genetically distant from other *Gephyrocapsa* species by genome sequences. Admixture pattern distinct from *G. pujosae* comb. nov. and *G. huxleyi* and forms the phylogenetic clade B (Fig. 1).

##### Basionym

*Emiliania pujosae* Verbeek 1990 p. 23-24 pl. 1 Figs. 4–9.

##### Synonym

*Emiliania huxleyi* var. *pujosae* Medlin & Green 1996 [29, 30].

##### Holotype

Metabolically inactivated strain PLY92D cryopreserved at the RCC as RCC174.

##### Habitat

Present in all oceans, temperate and high latitudes, found in water with monthly sea surface temperature ranging from 0 to 17 °C.

## Conclusion

This study demonstrates that the ecologically dominant coccolithophore *G. huxleyi*, also widely known as *E. huxleyi* and classically defined as a single species, is in fact composed of at least three genetically delineated species. Our genomic assessment reinforces the separation between A and B morphogroups, with the A morphogroup composing two of the main lineages. We also found further concordance between genotypic and morphotypic variability. Moreover, this complex is composed of diverse populations with restrictive preference in the environment. For instance, a broader sampling would help assess further the diversity of this interesting clade, and providing compiling evidence that more species could exist within this complex.

Our results reveal that the history of diversification in extant populations of *G. huxleyi* is restricted to the last 150 ka, likely driven by changes in habitat range due to contraction-expansion cycles of polar fronts linked to productivity cycles. We demonstrate that dominance of one species observed in the plankton fossil record corresponds to a rapid pulse of diversification of pseudo-cryptic species that adapted to local fluctuations of the environment. This evolutionary scenario provides insights into evolutionary links between biology and the environment, likely involving instances of hybridisation, which are relevant to the process that leads to the formation of cryptic and pseudo-cryptic species in keystone phytoplankton taxa.

The consequences of this pulsed diversification process in relation to the relevance of heavy and light calcifiers to global carbon sequestration remain to be assessed (cf. Fig. 4c). This could be addressed through detailed analysis of the sedimentary record (i.e., high-resolution sampling), a field in which metagenomic studies of sedimentary ancient DNA will become increasingly important. Despite the fact that understanding of the genetic basis of calcification is in its infancy [85], the present genomic comparison provides a robust framework to interpret future inter-strain physio-genomic comparisons aimed at understanding environmental influences on the biogeochemically key process of pelagic calcification.

## Supplementary information

Supplemental Material

## Data Availability

All sequences are available from NCBI under bioproject number PRJNA532411.
