# Supplementary material for: Rapid diversification underlying the global dominance of a cosmopolitan phytoplankton
Source: ISME J. 2023 Feb 6;17(4):630–40. doi: 10.1038/s41396-023-01365-5 (PMC10030636; doi:10.1038/s41396-023-01365-5)
Supplement: Supplementary file 1 — Supplemental Material [file 41396_2023_1365_MOESM1_ESM.docx]

**Rapid diversification underlying the global dominance of a cosmopolitan phytoplankton**

El Mahdi Bendif^1,2,3*^, Ian Probert^4^, Odysseas A Archontikis^1,5^, Jeremy R Young^6^, Luc Beaufort^7^, Rosalind E Rickaby^1^ and Dmitry Filatov^2^

^1^Department of Earth Sciences, University of Oxford, Oxford, UK

^2^Department of Plant Sciences, University of Oxford, Oxford, UK

^3^Institut des sciences de la mer de Rimouski (ISMER), Université du Québec à Rimouski, Rimouski, Canada

^4^Sorbonne Université – CNRS, Roscoff Culture Collection, FR2424 Station Biologique de Roscoff, Roscoff, France

^5^Department of Earth Sciences, The Natural History Museum, London, UK

^6^Department of Earth Sciences, University College London, London, UK

^7^Aix Marseille Université, CNRS, IRD, INRAE, Collège de France, CEREGE, Aix-en-Provence, France

*Corresponding author

**Email:**  [elmahdi.bendif@earth.ox.ac.uk](mailto:elmahdi.bendif@earth.ox.ac.uk)

**This PDF file includes:**

Supplementary Materials and Methods

Figures S1 to S6

Tables S1 to S7

SI References

**Supplementary Materials and Methods**

**Origin and morphological characterisation of analysed strains**

Of the 59 cultured strains of *G. huxleyi* included in our analysis, 47 strains characterized in this study were selected for an in-depth characterisation of genetic structure. This subset of strains represents several commonly found morphotypes in natural assemblages, with a majority of the morphogroup A including type A, over-calcified and R strains. The morphogroup B was represented by type B and O strains (Figure1, SI Fig. S1). Additionally, we included three non-calcified strains, which were isolated lacking coccoliths and recognised as *G. huxleyi* RCC1304, RCC1754 and RCC3746. Non-calcified cells reported in *G. huxleyi* and other *Gephyrocapsa* can be motile naked cells bearing two equal flagella as haploid cells as part of their haploid-diploid life cycle [1, 2]. Other notable non-motile naked cells (diploid) were reported in old cultures kept in stationary phase of growth but also in strains that have lost the ability to calcify before isolation such as RCC1754 or during long-term cultivation in culture collection as for CCMP1516 [3].

**Mapping**

Duplicated reads were removed using Picard (https://broadinstitute.github.io/picard/). The Genome Analyses ToolKit (GATK; https://gatk.broadinstitute.org/hc/en-us) was then used for base quality recalibration, local realignment around Indels (insertions/deletions). SNPs (Single Nucleotide Polymorphisms) calling was performed using SAMTOOLS v1.2 [4]. Positions covered by at least eight reads were retained after removing reads with mapping quality below 20 (−q 20) and bases with base quality below 20 (−Q 20) to compensate for variant retrieval in strains with mapping coverage below 20x (https://gatk.broadinstitute.org/hc/en-us). Homozygous-reference regions with a minimum depth of eight reads (−g 8) were included, but we excluded single-nucleotide polymorphisms (SNPs) within 3 bp of an indel (−g 3). SNPs with quality below 15 were further filtered out as well as heterozygous SNPs with fewer than two reads supporting each allele. These obtained SNPs in vcf file format were converted into fasta multiple alignment sequences per contig (7,795 contigs) using a custom script *vcf2fas* (available at https://github.com/brunonevado/vcf2fas) for the 59 genomes of *G. huxleyi* available.

**Phylogenetic reconstructions**

For the dataset comprising 47 strains of *G. huxleyi*, we retained 430 contigs with no individual missing from the 7,795 contigs. Contigs were then split in distinct regions of 5 kb long separated by 25 kb regions retained randomly 1,000 supergenes excluding the alignment positions with >20% of gaps. We first reconstructed a species phylogeny using a multi-species coalescent-based approach accounting for incomplete lineage sorting (ILS). We performed ML reconstructions for all the 1,000 supergenes using the GTRGAMMA model and 100 bootstrap replicates in RAxML 8 [5]. BestML trees were then used to produce a species tree using ASTRAL [6]. We also inferred a ML tree based on the concatenation of the 1,000 supergenes forming a matrix of 5 Mb of which 306.5 kb were phylogenetically informative using the GTRGAMMA model and 100 bootstrap replicates.

For the dataset comprising 59 strains of *G. huxley,* we retained 197 contigs from the 7,795 with no individual missing and around 80% data completion. Contigs were then split in distinct alignments of 5 kb long for each 25 kb window ending up with 829 supergene-alignments (4,145 kb). For each of these 829 alignments, we performed a Maximum likelihood (ML) phylogenetic reconstruction using the GTRGAMMA model and 100 bootstrap replicates in RAxML 8 [5]. BestML trees with bootstrap replicates were then used to produce a species tree using ASTRAL [6]. We also inferred a ML phylogenetic tree based on the concatenation of the 829 contigs forming a matrix of 4.15 Mb of which 103.78 kb were phylogenetically informative with RAxML using the GTRGAMMA model and 100 bootstrap replicates.

**Gene flow analysis**

We performed a Patterson’s *D*-statistic test which compares two phylogenetically incongruent site patterns of ancestral (A) and derived (B) alleles ABBA—(((A,B),B),A) and BABA—(((B,A),B),A) on a four-taxon phylogeny with the topology: (((P1,P2),P3),Outgroup). If the incongruence is due to ILS, the frequencies of these site patterns are expected to be equal, but in the case of gene flow between P3 and either P1 or P2, they are expected to be biased toward the site pattern that clusters the admixed taxa together. Thus, gene flow between P2 and P3 will lead to an excess of ABBA patterns associated to a positive D-statistic, a surplus excess of BABA patterns would reflect a negative D-statistic suggestive of gene flow between P1 and P3. In the Dsuite package, D-statistic are always positive as P1 and P2 are ordered. Z-scores and associated p-values were calculated by block-jackknife procedure to assess the significance for a deviation of the D-statistic from zero. P-values were adjusted by the Benjamin-Hochberg correction with p-value < 0.05 as an indication for potential signal of gene flow [10]. *F4 ratios* were calculated along to provide an estimate of the excess of allele shared between the trios of species tested. *Fbranch* (*f_b_*), branch specific statistics, were then calculated to summarize and validate the significance of the gene flow events measured. In this analysis, we merged vcf files of *Gephyrocapsa oceanica* strains retrieved from the read mapping in [9] to our current dataset of 47 strains to have an outgroup using the *merge* option in the BCFtools program [4].

**Divergence time**

To the 57 strains dataset, we added strains of *G. muellerae* (1) and *G. ericsonii/parvula* (3) as outgroup to vcf files, prior to fasta conversion and performed a downstream filtration as described for phylogenetic analyses. We conducted two separate runs of 100 million generations, with samples taken every 1,000 generations, using the uncorrelated lognormal relaxed clock model under the GTR+I+G model of evolution determined by ModelTest-NG [7]. A maximum clade credibility tree was obtained using Tree Annotator within the BEAST software package with a burn-in of 25 % trees. Acceptable sample sizes and convergence to the stationary distribution were checked using Tracer 1.7 [8]. The [molecular clock](https://www.sciencedirect.com/topics/biochemistry-genetics-and-molecular-biology/molecular-clock) was calibrated using the origin of *G. huxleyi* in the fossil record estimated at 291 ka, representing the split between *G. huxleyi* branch and other *Gephyrocapsa* as in previous study [9].

**Demographic modelling**

For these inferences, we used the reduced dataset used to infer the phylogenetic network (85,365 SNPs). For all models, we performed consecutive rounds of optimizations. For each round, we ran multiple replicates and used parameter estimates from the best scoring replicate (highest log-likelihood) to seed searches in the following round. We used the default settings in dadi_pipeline for each round (replicates = 10, 20, 30, 40; maxiter = 3, 5, 10, 15; fold = 3, 2, 2, 1), and optimized parameters using the Nelder-Mead method (optimize_log_fmin). Across all analyses, we used the optimized parameter sets of each replicate to simulate the 2D-JSFS, and the multinomial approach was used to estimate the log-likelihood of the 2D-JSFS given the model. Parametric bootstrap function (via ‘‘.sample’’ function in dadi) was used to estimate the bootstrap confidence intervals for the parameter estimates for the best-fitting models, based on 100 bootstrap replicates. Coalescent parameters were converted following [11]. Parameter of ancestral effective population size (*N_ref_*) was calculated as *N_ref_* = *θ*/4*µl*, where *θ* was the scaled population parameter, *µ* was the mutation rate per site and per generations estimated in [12], and *l* the length of analysed sequence. Migration was calculated as *m* = *M*/2*N*_ref_ and time in years as *t* = 2*TN*_ref_ × *g*, using a generation time of 50 division per year as in [11].

**Environmental data and related statistical analysis**

Regarding strains isolated before 1981 for which satellite data are not available, we used the seasonal average temperature at the isolation site (grid of 5° squares) since 1985 as for strains for which isolation date are not available. Uncorrelated variables (*r* < 0.6) were retained in order to avoid testing strongly interdependent models and to effectively estimate the relative importance of different factors. This resulted in 4 main predicators to test (sea surface temperature, surface phosphate concentration, sea surface pH, and dissolved inorganic carbon (DIC)). Redundancy analysis (RDA) [13] was used to study genotype–environment associations using the *vegan* package [14] in R. Genetic and environmental data were analysed using multivariate linear regression, producing a matrix of fitted values (i.e., constrained axes) and then a principal component analysis (PCA) of the fitted values produces canonical axes (i.e., unconstrained axes), which are linear combinations of the predictors. A permutation test was performed with 999 permutations using the function *anova.cca()* in order to assess significance for the RDA. At last, we performed a Dunn’s test for stochastic dominance to compare the distribution of each phylogenetic clades/sub-clades according to environmental parameters using the R package *dunn.test*. We reported the results among multiple pairwise comparisons after a Kruskal-Wallis test for stochastic dominance among k groups [15] and significance with *p*-values corrected using Bonferroni method.


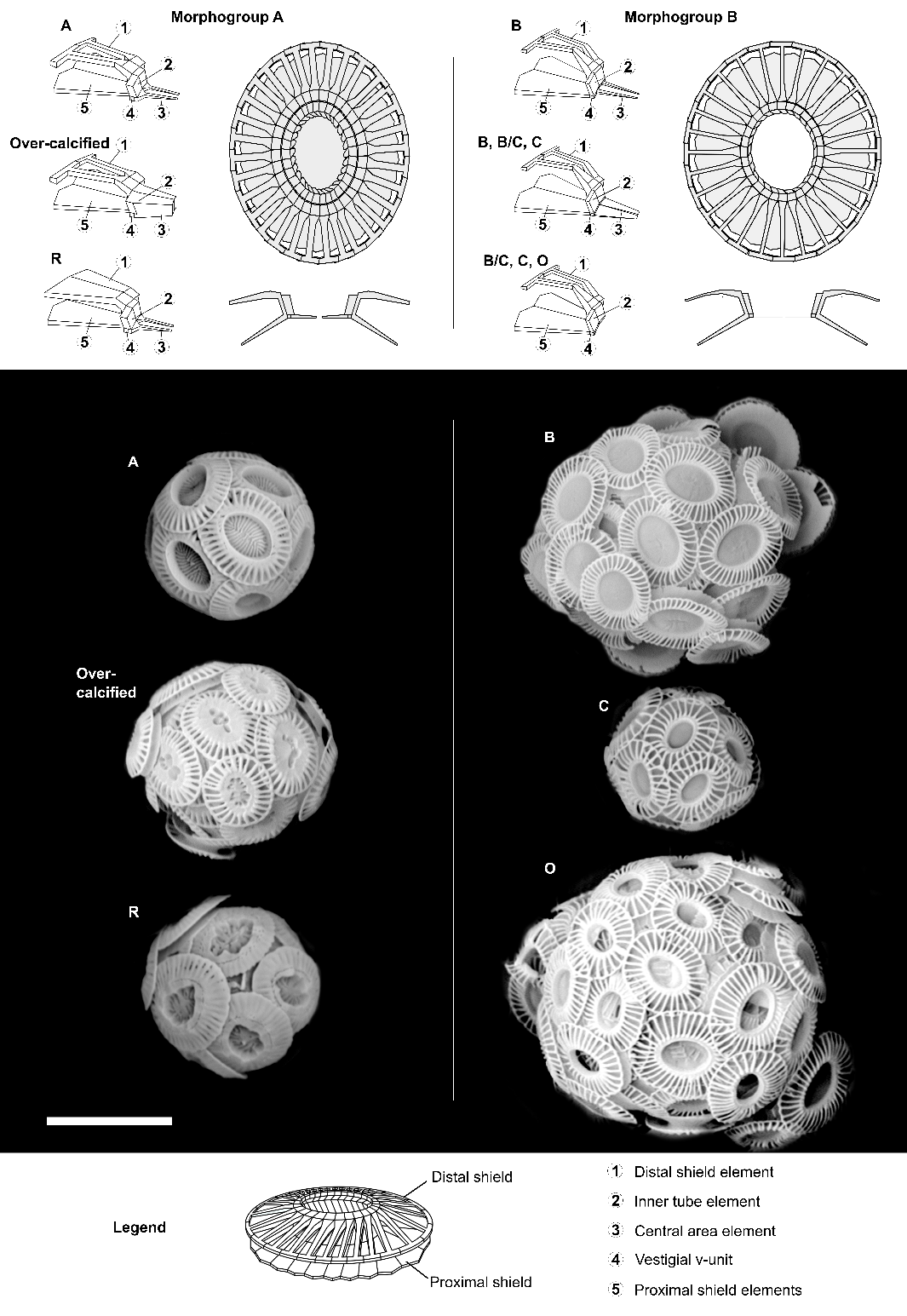


**Fig. S1**. Classification of morphotypes in *G. huxleyi*. Left panel shows major features of the morphogroup A with commonly found morphotypes (A, over-calcified and R) compared to the right panel showing morphogroup B with associated morphotypes (B, C and O). Scale bar = 4 μm.


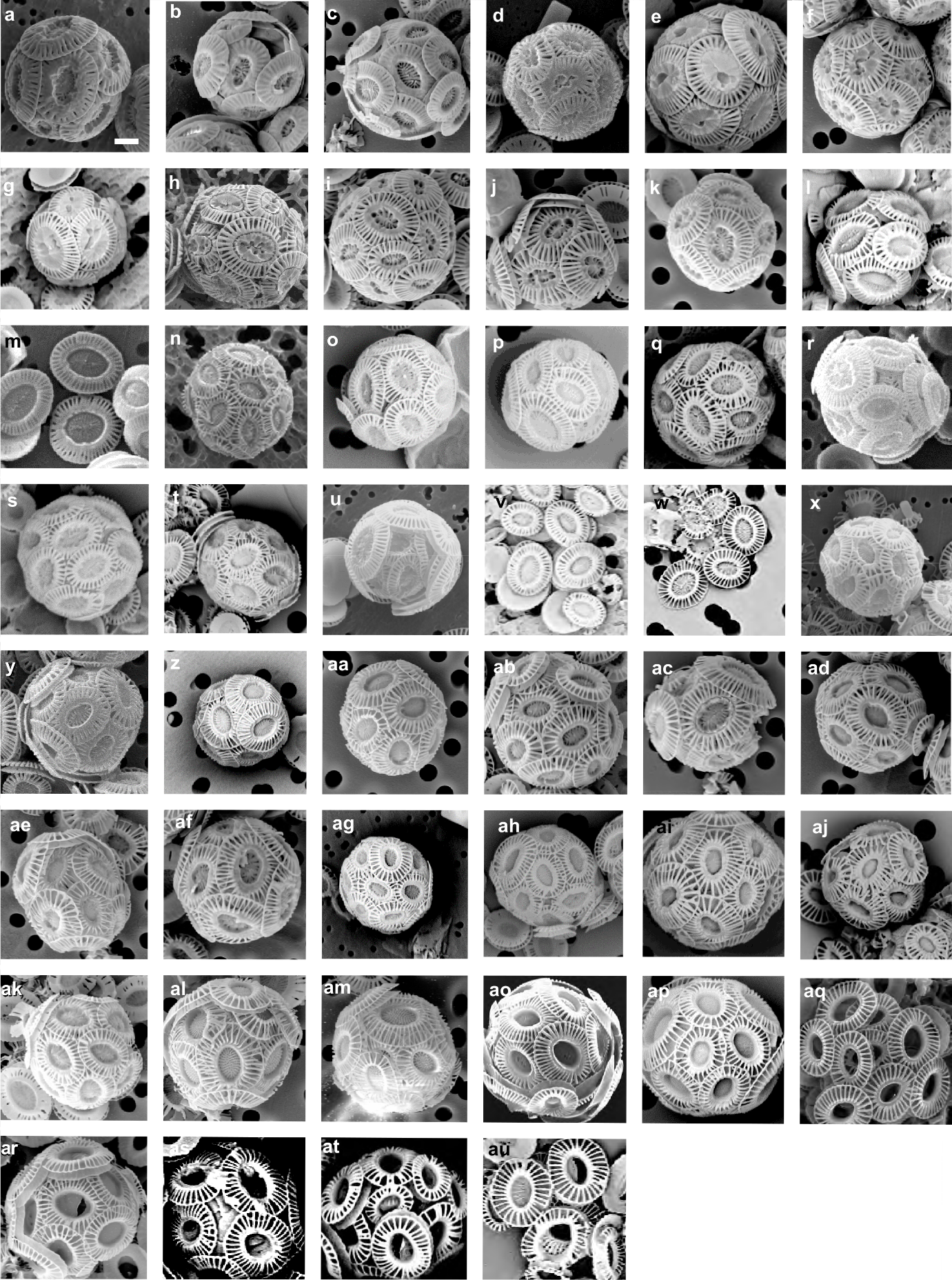


**Fig. S2**: Scanning electron micrographs (Scanning Electron Microscopy) of *G. huxleyi* strains used in this study. Strain correspondence is provided in supplementary table 2. All micrographs are at the same scale (scale bar = 1 μm).


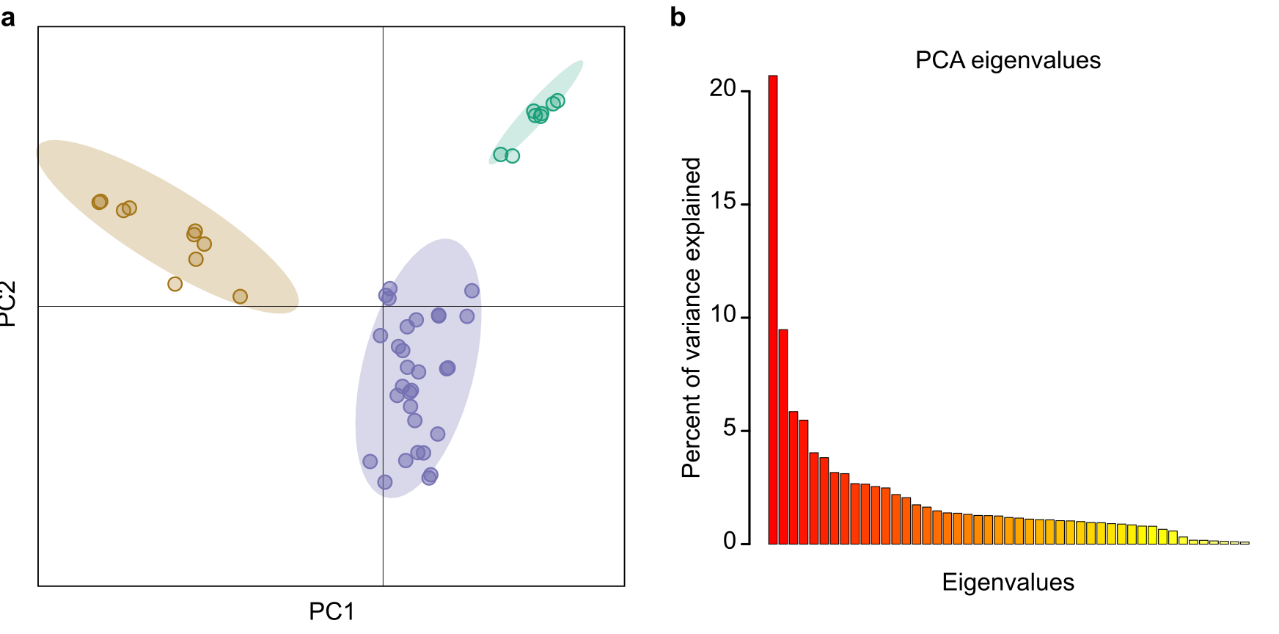


**Fig. S3**: a. Principal component analysis (PCA) based on 2,086,643 SNPs recovered from 47 *G. huxleyi* genomes, b. Amount of variance that can be explained by each principal component in the PCA.


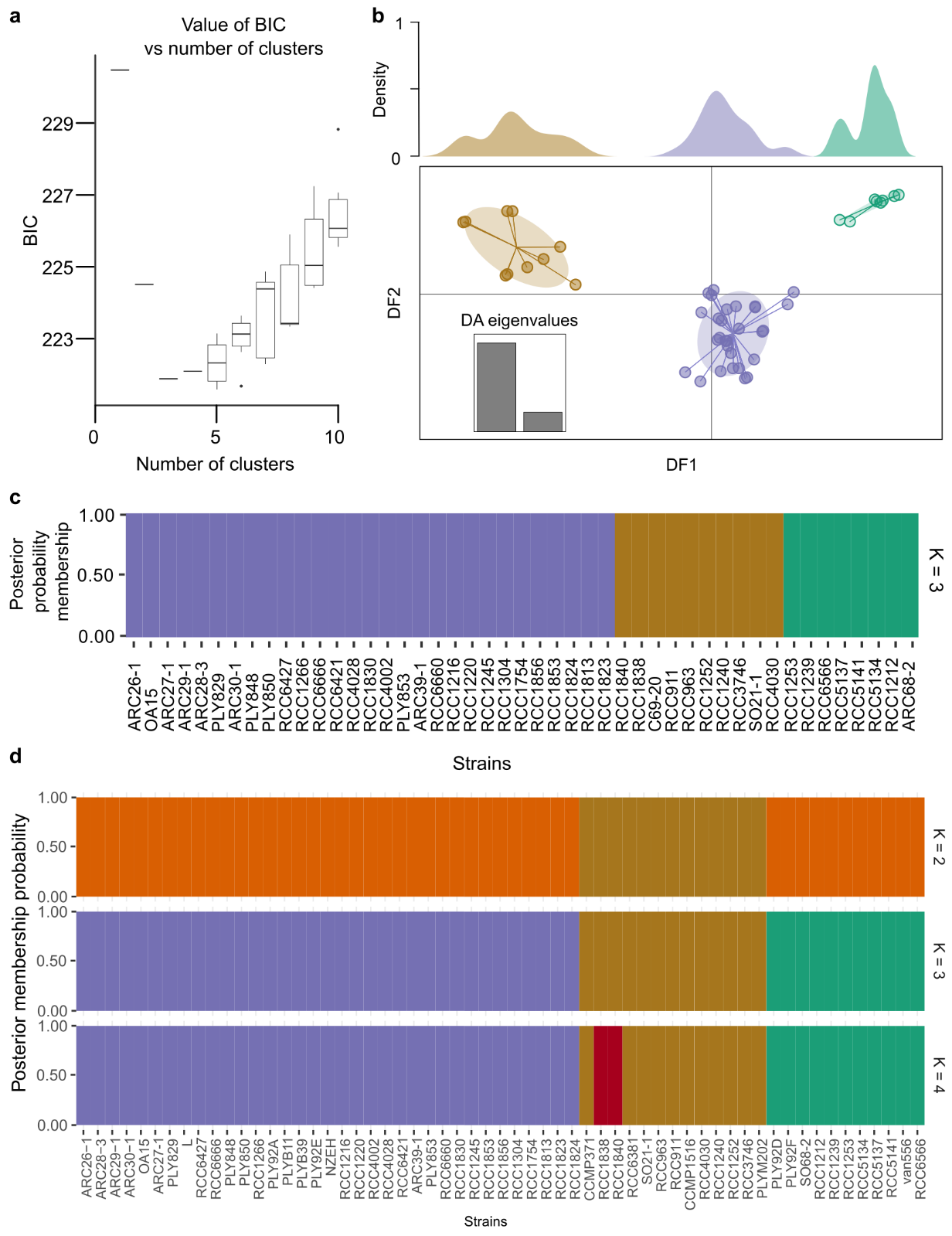


**Fig. S4**: Discriminant analysis in principal component (DAPC) based on 2,086,643 SNPs recovered from 47 (a-c) and 59 (d) *G. huxleyi* genomes, a. Selection of optimal cluster for the DAPC using successive k-means and the lowest Bayesian Information Criterion (BIC), b. DAPC plot with density plot for the discriminant factor 1 (DF1), c. Chromplot of the DAPC for 3 clusters (K=3), d. Chromplots of DAPC recovered from 59 G. huxleyi genomes (including low covered genomes).


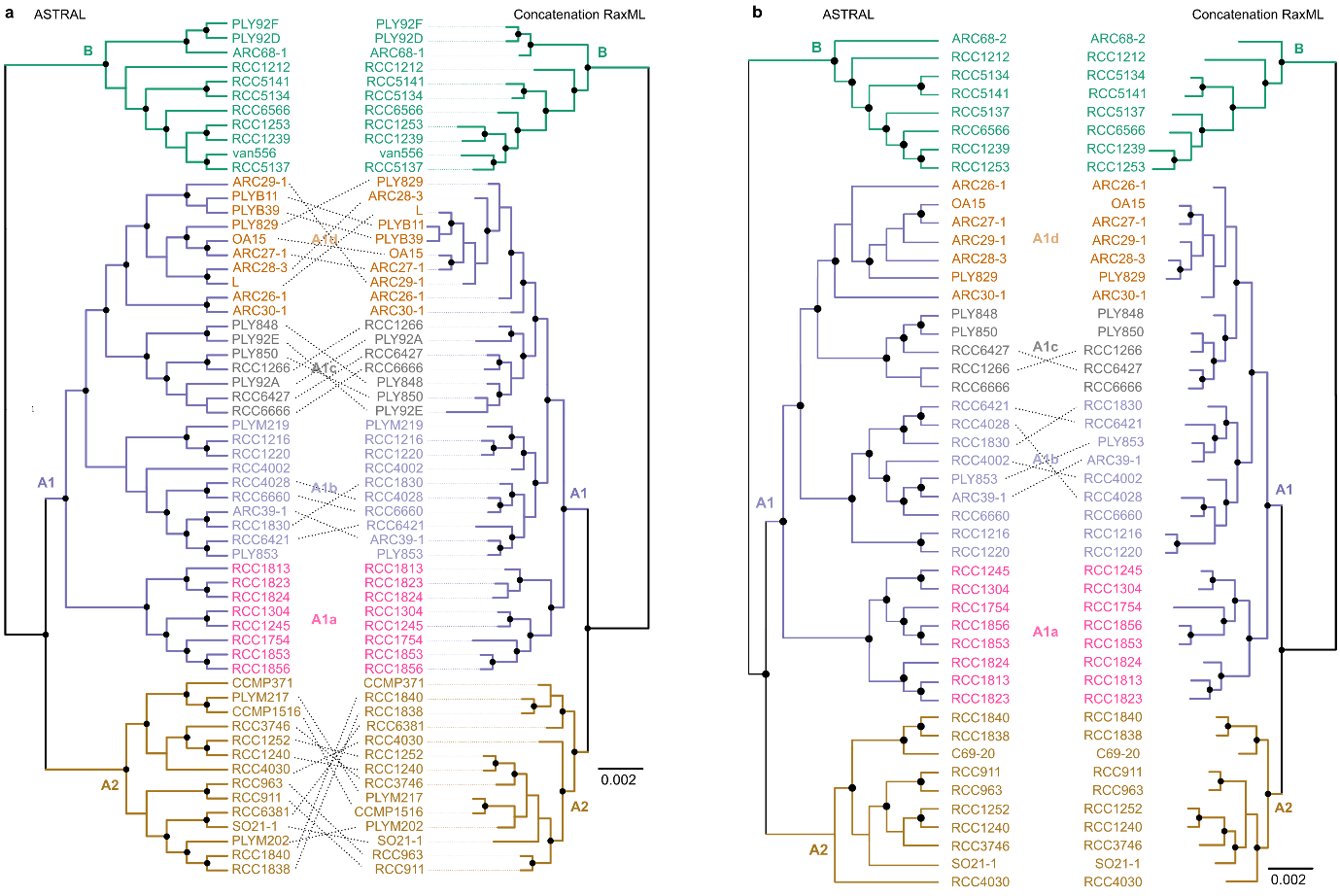


**Fig. S5:** Phylogenetic relationships between *G. huxleyi* strains, a. Coalescent and concatenated phylogenies based on 59 strains of *G. huxleyi*, and respectively 829 supergenes of 10 kb and an alignment of 4.15 Mb, b. Coalescent and concatenated phylogenies based on 47 strains of *G. huxleyi*, and respectively 1000 supergenes of 10 kb and an alignment of 5 Mb. Dotted lines represent topological incongruences.


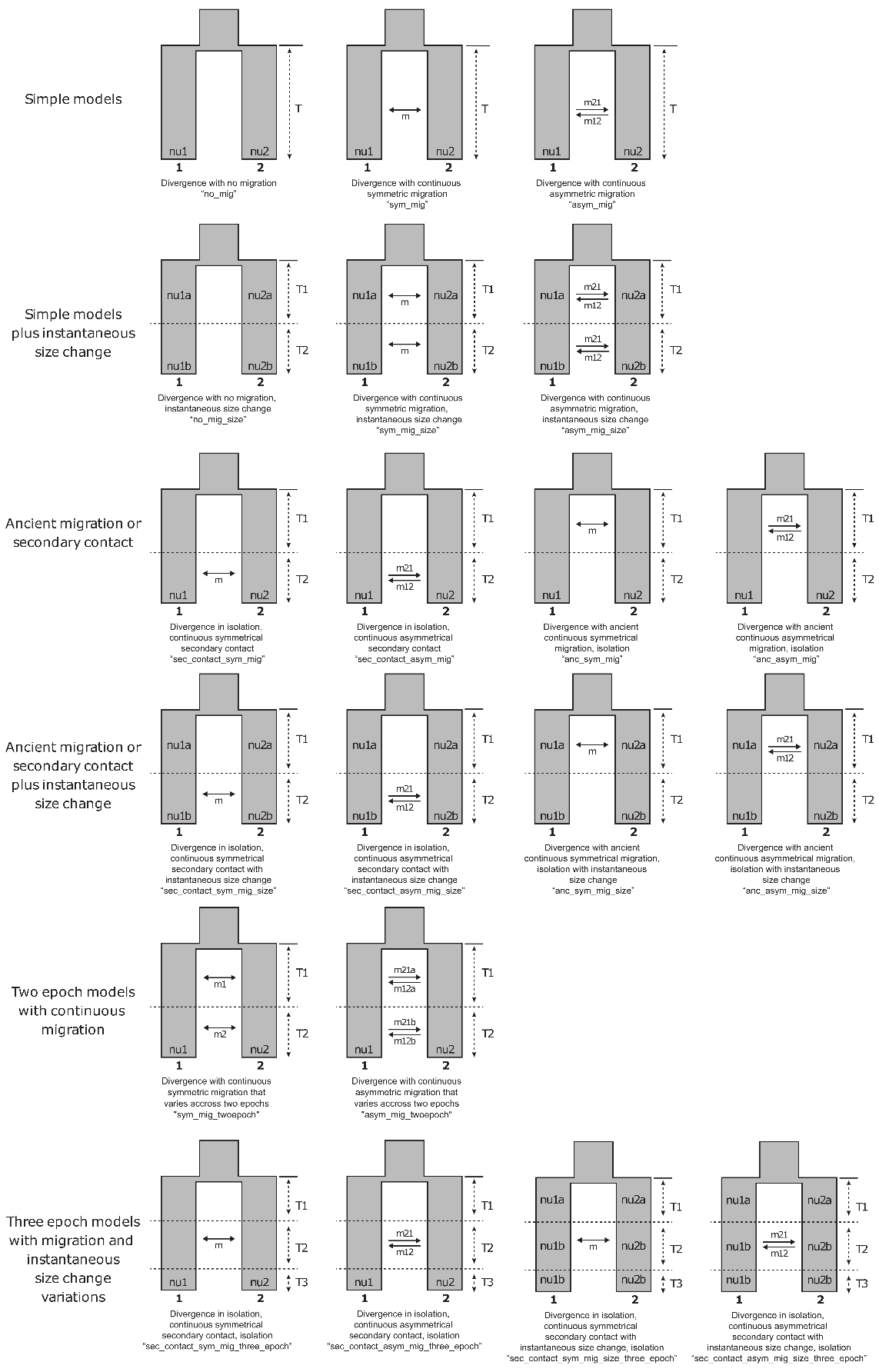


**Fig. S6**: Parameters in the 20 models analysed[16]. m: symmetrical migration rate between species, m12: asymmetrical migration rate from species 1 to species 2, m21: asymmetrical migration rate from species 2 to species 1, nu1: effective population size of extant species 1, nu1a: effective population size of extant species 1 between speciation and the beginning of the second phase, nu1b: effective population size of extant species 1 during the second phase, nu2: effective population size of extant species 2, nu2a: effective population size of extant species 1 between speciation and the beginning of the second phase, nu2b: effective population size of extant species 1 during the second phase, T and T1: time of speciation, T2: duration of the second phase in two epoch models, T3: duration of the third phase in three epoch models.

**Table S1** Classification of morphotypes of *Gephyrocapsa huxleyi*.

| **Morphogroup** | **Morphotype** | **Central grill** | **Typical coccolith size** | **Inner tube** | **Distal shield elements** | **Distal shield profile** | **Proximal shield width** | **Author** | **other names** | **Biogeography** |
| --- | --- | --- | --- | --- | --- | --- | --- | --- | --- | --- |
| A | R | curved laths or obscured | 3-4 µm | Thick | very thick | flat | always narrower than distal shield | [17] | *var. huxleyi* | global |
| A | A overcalcified | curved laths or obscured | 3-4 µm | Thick and very thick (can close central aera) | variable | flat | always narrower than distal shield | [17] |  | global |
| A | A | curved laths | 3-4 µm | narrow to moderate | variable | flat | always narrower than distal shield | [18] |  | global |
| B | B | irregular laths | 4-5 µm | narrow | narrow | curved | often wider than distal shield | [18] | *var. pujosae* | N Atlantic higher latitudes |
| B | B/C | irregular laths or open | 3-4 µm | narrow to moderate | narrow | curved | often wider than distal shield | [17] | *var. aurorae* | Southern ocean |
| B | C | irregular laths or open | 2-3 µm | narrow | narrow | curved | often wider than distal shield | [18] | *var. kleijniae* | Higher latitudes |
| B | O | open | 4-5 µm | narrow | narrow | curved | often wider than distal shield | [19] |  | Pacific higher latitudes |

**Table S2** List of samples used in the analyses. AC: Algobank Caen; CCMP: Bigelow National centre for Marine Algae and Microbiota; PLY: Plymouth culture collection; RCC: Roscoff Culture Collection.

| Strain ID | Other names |  | Region  /Ocean | Latitude | Longitude | Isolator | Date of isolation | Morphogroup | Morphotype | SEM picture* | Remark |
| --- | --- | --- | --- | --- | --- | --- | --- | --- | --- | --- | --- |
| ARC26-1 |  |  | Greenland Sea | 79.20 | 0.00 | C. Balestreri | July 2012 | A | A | o |  |
| ARC27-1 |  |  | North Greenland | 78.51 | -6.14 | C. Balestreri | July 2012 | A | A | r |  |
| ARC28-3 |  |  | Greenland Sea | 78.41 | -1.49 | C. Balestreri | July 2012 | A | A | p |  |
| ARC29-1 |  |  | Greenland Sea | 78.70 | 3.28 | C. Balestreri | July 2012 | A | A | m |  |
| ARC30-1 |  |  | Western Coast of Svalbard | 79.00 | 8.00 | C. Balestreri | July 2012 | A | A | l |  |
| ARC39-1 |  |  | North Greenland | 76.30 | -2.92 | C. Balestreri | July 2012 | A | overcalcified | d |  |
| ARC68-2 |  |  | Northern Icelandic Sea | 67.83 | -12.20 | C. Balestreri | July 2012 | B | B | ao |  |
| CCMP1516 | RCC1242 |  | Pacific Ocean | -2.67 | -82.71 |  | 1991 |  |  |  | Now naked |
| CCMP371 | 12-1 |  | Atlantic Ocean | 32.00 | -62.00 | B. Palenik | 1987 | A | A | see |  |
| L |  |  | Oslo Fjord | 60.00 | 11.00 |  | 1959 | A | A | see[18] | culture lost |
| OA15 |  |  |  |  |  | C. Balestreri |  |  |  |  | culture lost |
| PLY829 | D366 36-2 |  | North Sea | 56.50 | 3.65 | C. Balestreri | July 2011 | A | A | v |  |
| PLY848 | D366 71-4 |  | Bay of Biscay | 46.20 | -7.21 | C. Balestreri | June 2011 | A | A | t |  |
| PLY850 | D366 80-1 |  | Bay of Biscay | 45.70 | -7.16 | C. Balestreri | June 2011 | A | A | w |  |
| PLY853 | D366 80-4 |  | Bay of Biscay | 45.70 | -7.16 | C. Balestreri | July 2011 | A | overcalcified | g |  |
| PLY92A | 92A | CCMP379 | English Channel | 50.17 | -4.25 | I. Parke | 1957 | A | A | see[18] | Now naked |
| PLY92D | 92D |  | English Channel | 50.03 | -4.37 | J. C. Green | 1975 | B | B[18] | see[18] | Now naked |
| PLY92E | 92E |  | English Channel | 49.87 | -6.20 | J. Green | 1992 |  |  |  | Now naked |
| PLY92F | 92F |  | English Channel | 49.87 | -6.20 | J. Green | 1992 |  |  |  | Now naked |
| PLYB11 | B11 |  | Bergen Fjord | 60.12 | 5.23 | J. Green | 1992 | A | A |  |  |
| PLYB39 | B39 |  | Bergen Fjord | 60.12 | 5.23 | J. Green | 1992 | A | A |  |  |
| PLYM202 | EH2 |  | South Pacific | -21.00 | 151.00 | I. Inouye | 1990 | A | A |  | Now naked |
| PLYM217 | M217, CCMP1516 | RCC1731 | Pacific Ocean | -2.67 | -82.71 |  | 1991 | A | A | aj |  |
| PLYM219 | NZEH |  | South Pacific | -47.00 | 168.00 | L. Rhodes | 1992 | A | R | a |  |
| NZEH | M219 |  | South Pacific | -47.00 | 168.00 | L. Rhodes | 1992 | A | R | a |  |
| RCC1212 | NS10Y | AC477 | Atlantic Ocean | -34.50 | 17.30 | I. Probert | September 2000 | B | B | ap |  |
| RCC1216 | TQ26 | AC472 | Pacific Ocean | -42.30 | 169.83 | I. Probert | September 1998 | A | R | c |  |
| RCC1220 | TQ22 | AC468 | Pacific Ocean | -42.30 | 169.83 | I. Probert | September 1998 | A | R | b |  |
| RCC1239 | OS5 | AC674 | Pacific Ocean | 43.22 | 141.02 | K. Hagino | April 2006 | B | O | aq |  |
| RCC1240 | MT0610A | AC676 | Pacific Ocean | 41.50 | 141.25 | K. Hagino | October 2006 | A | A |  |  |
| RCC1245 | LK6 | AC298 | Atlantic Ocean | 45.00 | -1.08 | I. Probert | February 1999 | A | A | n, q |  |
| RCC1252 | MT0610E | AC678 | Pacific Ocean | 41.50 | 141.25 | K. Hagino | October 2006 | A | A | ad |  |
| RCC1253 | OS2 | AC675 | Pacific Ocean | 43.22 | 141.02 | K. Hagino | April 2006 | B | O | ar |  |
| RCC1266 | BG10-5 |  | Celtic Sea | 49.50 | -10.50 | I. Probert | 2008 | A | A | s |  |
| RCC1304 | PC71 | AC296 | Atlantic Ocean | 38.20 | -9.63 | I. Probert | October 1998 |  |  |  | Now naked |
| RCC1754 | RE070718_25-4 |  | English Channel | 48.75 | -3.95 | F. Le Gall | 2007 |  |  |  | Isolated naked |
| RCC1813 | BOUM12 |  | Mediterranean Sea | 33.63 | 32.65 | I. Probert | June 2008 | A | A | ak |  |
| RCC1823 | BOUM22 |  | Mediterranean Sea | 33.63 | 32.65 | I. Probert | June 2008 | A | A | af |  |
| RCC1824 | BOUM23 |  | Mediterranean Sea | 33.63 | 32.65 | I. Probert | June 2008 | A | A | ab |  |
| RCC1830 | BOUM39 |  | Mediterranean Sea | 39.10 | 5.35 | I. Probert | July 2008 | A | overcalcified | h |  |
| RCC1838 | BOUM48 |  | Mediterranean Sea | 39.10 | 5.35 | I. Probert | July 2008 | A | A | ae |  |
| RCC1840 | BOUM51 |  | Mediterranean Sea | 39.10 | 5.35 | I. Probert | June 2008 | A | A | ac |  |
| RCC1853 | BOUM81 |  | Mediterranean Sea | 34.13 | 18.45 | I. Probert | June 2008 | A | A | al |  |
| RCC1856 | BOUM73 |  | Mediterranean Sea | 34.13 | 18.45 | I. Probert | June 2008 | A | A | am |  |
| RCC3746 | TMR1 |  | Pacific Ocean | 35.87 | 134.57 | K. Hagino | June 2007 | A | A | x |  |
| RCC4027 | CHC307 |  | Juan Fernandez | -33.63 | -78.82 | C. Henriquez | October 2011 | A | overcalcified | i |  |
| RCC4028 | CHC350 |  | Pacific Ocean | -30.25 | -71.70 | C. Henriquez | October 2013 | A | overcalcified | e |  |
| RCC4030 | CHC428 |  | Pacific Ocean | -16.70 | -86.00 | C. Henriquez | November 2013 | A | A | ai |  |
| RCC5134 | CHC548 |  | Pacific Ocean | -30.15 | -71.97 | H. A. Ramírez | November 2015 | B | O | as |  |
| RCC5137 | CHC576 |  | Pacific Ocean | -30.15 | -71.97 | H. A. Ramírez | November 2015 | B | O | at |  |
| RCC5141 | CHC579 |  | Pacific Ocean | -30.15 | -71.97 | H. A. Ramírez | November 2015 | B | O | au |  |
| RCC6381 | C69-20 |  | Atlantic Ocean | 27.98 | -15.37 | I. Probert | February 2014 | A | A | ah |  |
| RCC6421 | J1 |  | Celtic Sea | 52.46 | -5.90 | I. Probert | June 2011 | A | overcalcified | j |  |
| RCC6427 | J8 |  | Celtic Sea | 52.46 | -5.90 | I. Probert | June 2011 | A | A | u |  |
| RCC6566 | P17-018 |  | Pacific Ocean | 37.60 | 121.60 | I. Probert | Mars 2016 |  |  |  | Now naked |
| RCC6660 | P41 |  | Pacific Ocean | -14.03 | -76.28 | I. Probert | 2014 | A | overcalcified | f |  |
| RCC6666 | P46 |  | Pacific Ocean | -14.17 | -76.63 | I. Probert | 2014 | A | A | k |  |
| RCC911 |  |  | Pacific Ocean | -8.33 | -141.25 | D. Vaulot, D. Marie | November 2004 | A | A | ag |  |
| RCC963 |  |  | Pacific Ocean | -8.33 | -141.25 | D. Vaulot, D. Marie | November 2004 | A | A | z |  |
| SO 21-1 |  |  | SE South Africa | -38.31 | 40.96 | C. Balestreri | April 2013 | A | A | y |  |
| Van556 |  |  | Pacific Ocean | 49.00 | -145.00 |  | 1984 |  |  |  | culture lost |

* cf. Fig. S2.

**Table S3** List of genomic read samples used in the analyses.

| Strain ID | Length of mapping coverage (%) | Breadth of mapping coverage | Genbank accession number | Reference |
| --- | --- | --- | --- | --- |
| ARC26-1 | 66.6647 | 84.184503 | SRR17818300 | This study |
| ARC27-1 | 60.87421 | 59.331124 | SRR14251530 | ^5^ |
| ARC28-3 | 62.19828 | 46.831551 | SRR17818299 | This study |
| ARC29-1 | 65.57376 | 114.73994 | SRR17818288 | This study |
| ARC30-1 | 64.07297 | 64.301737 | SRR8885247 | ^6^ |
| ARC39-1 | 63.66436 | 41.688662 | SRR14251529 | ^5^ |
| ARC68-2 | 48.92304 | 21.412445 | SRR14251528 | ^5^ |
| CCMP1516 | 43.10898 | 19.488725 | SRR391471 | ^7^ |
| CCMP371 | 29.80708 | 19.45506 | SRR391477 | ^7^ |
| L | 27.34222 | 16.842095 | SRR391476 | ^7^ |
| OA15 | 60.76794 | 58.233862 | SRR14251540 | ^5^ |
| PLY829 | 56.72082 | 64.016086 | SRR14251552 | ^5^ |
| PLY848 | 59.98016 | 76.797368 | SRR17818274 | This study |
| PLY850 | 47.45776 | 64.054321 | SRR17818273 | This study |
| PLY853 | 61.64081 | 54.064974 | SRR14251551 | ^5^ |
| PLY92A | 24.81075 | 17.676571 | SRR391474 | ^7^ |
| PLY92D | 21.506 | 18.470797 | SRR391472 | ^7^ |
| PLY92E | 25.91082 | 19.529685 | SRR391473 | ^7^ |
| PLY92F | 22.87558 | 19.057228 | SRR391484 | ^7^ |
| PLYB11 | 28.95966 | 15.485682 | SRR391480 | ^7^ |
| PLYB39 | 28.70285 | 15.7393 | SRR391481 | ^7^ |
| PLYM202 | 26.73826 | 17.623158 | SRR391478 | ^7^ |
| PLYM217 /CCMP1516 | 47.22137 | 17.974514 | SRR391482 | ^7^ |
| PLYM219 /NZEH | 27.21425 | 16.635266 | SRR391475 | ^7^ |
| PLYM219 /NZEH | 28.75605 | 14.573299 | SRR391479 | ^7^ |
| RCC1212 | 60.70548 | 21.439867 | SRR14251548 | ^5^ |
| RCC1216 | 61.7059 | 72.691161 | SRR17818272 | This study |
| RCC1220 | 48.26211 | 70.890969 | SRR17818298 | This study |
| RCC1239 | 53.96939 | 54.372241 | SRR14251525 | ^5^ |
| RCC1240 | 53.58004 | 72.834472 | SRR17818297 | This study |
| RCC1245 | 65.54055 | 78.574977 | SRR17818296 | This study |
| RCC1252 | 61.89828 | 62.816291 | SRR17818295 | This study |
| RCC1253 | 46.92411 | 90.867466 | SRR8885248 | ^6^ |
| RCC1266 | 60.57517 | 26.360055 | SRR17818294 | This study |
| RCC1304 | 60.87509 | 69.846918 | SRR17818293 | This study |
| RCC1754 | 52.37383 | 27.759848 | SRR17818292 | This study |
| RCC1813 | 64.36075 | 98.596093 | SRR17818291 | This study |
| RCC1823 | 61.63835 | 89.655626 | SRR17818290 | This study |
| RCC1824 | 63.98082 | 47.000144 | SRR17818289 | This study |
| RCC1830 | 53.06351 | 36.119069 | SRR17818287 | This study |
| RCC1838 | 49.67896 | 71.710819 | SRR17818286 | This study |
| RCC1840 | 52.72371 | 43.267234 | SRR17818285 | This study |
| RCC1853 | 60.78892 | 79.480847 | SRR17818284 | This study |
| RCC1856 | 59.89225 | 47.714944 | SRR17818283 | This study |
| RCC3746 | 56.69525 | 69.830421 | SRR17818282 | This study |
| RCC4027 | 58.95349 | 68.911927 | ERR695588 | ^8^ |
| RCC4028 | 61.516 | 138.8515 | ERR695589 | ^8^ |
| RCC4030 | 61.21289 | 112.92009 | ERR695590 | ^8^ |
| RCC5134 | 62.0252 | 52.882475 | SRR14251550 | ^5^ |
| RCC5137 | 62.20505 | 63.272983 | SRR14251527 | ^5^ |
| RCC5141 | 52.63836 | 57.954924 | SRR14251526 | ^5^ |
| RCC6381 | 52.94296 | 50.317507 | SRR17818279 | This study |
| RCC6421 | 66.54316 | 23.078814 | SRR17818278 | This study |
| RCC6427 | 59.13884 | 37.338684 | SRR17818277 | This study |
| RCC6566 | 54.48605 | 27.134271 | SRR14251549 | ^5^ |
| RCC6660 | 66.32684 | 33.107018 | SRR17818276 | This study |
| RCC6666 | 61.19155 | 28.875358 | SRR17818275 | This study |
| RCC911 | 59.58616 | 31.671522 | SRR17818281 | This study |
| RCC963 | 61.15752 | 33.559535 | SRR17818280 | This study |
| SO 21-1 | 61.74681 | 49.379293 | SRR14251531 | ^5^ |
| Van556 | 23.48322 | 19.581546 | SRR391483 | ^7^ |

**Table S4**: Correspondence between genotypes and morphotype descriptions

| Genotype | Morphogroup | Morphotype | Inner tube | Grill | Distal shield elements | Distal shield profile | Proximal shield width | Biogeography in this study |
| --- | --- | --- | --- | --- | --- | --- | --- | --- |
| A1a | A | A | narrow, moderate | thieve-like grid with regular curved rods, curved laths | narrow | flat | always narrower than distal shield | low latitude |
| A1b | A | A overcalcified, R | very broad, broad | curved laths or obscured | variable, very thick | flat | always narrower than distal shield | global |
| A1c | A | A | narrow with irregularities | curved laths | moderate, thick | flat | always narrower than distal shield | transitional |
| A1d | A | A | narrow with irregularities | curved laths | moderate | flat | always narrower than distal shield | high latitudes (Atlantic ocean) |
| A2 | A | A | narrow, broad | Irregular laths, moderately calcified | narrow | flat | always narrower than distal shield | low latitudes |
| B | B | B, O | narrow | light laths, open | narrow | curved | often wider than distal shield | Atlantic, Pacific (both high latitudes) |

**Table S5**: The D-statistic and f4-ratio tests for interspecific and intraspecific gene-flow between 3 *Gephyrocapsa* species, with *G. oceanica* as an outgroup (not shown). The first row corresponds to interspecific comparison. * D-statistic values for each of the species quartets. ** Z-score showing D-statistic in units of its standard deviation, with Z > 3 can be regarded as significantly different from zero. *** Bonferroni-adjusted P-value for significant difference from zero. **** f4-ratio showing an estimated proportion of interspecific admixture. Rows with the most significant values are in bold.

| Species 1 | Species 2 | Species 3 | Dstatistic* | Z-score** | p-value*** | f4-ratio**** |
| --- | --- | --- | --- | --- | --- | --- |
| A1 | A2 | B | 0.001 | 0.559 | 0.288 | 0.021 |
| **A1a** | **A1b** | **B** | **0.017** | **9.031** | **0.000** | **0.197** |
| **A1a** | **A1c** | **B** | **0.029** | **15.417** | **0.000** | **0.378** |
| **A1a** | **A1d** | **B** | **0.036** | **17.913** | **0.000** | **0.423** |
| **A1a** | **A2** | **B** | **0.018** | **6.660** | **0.000** | **0.222** |
| **A1b** | **A1c** | **A1a** | **0.026** | **18.137** | **0.000** | **0.414** |
| **A1b** | **A1c** | **B** | **0.013** | **6.185** | **0.000** | **0.191** |
| **A1b** | **A1d** | **A1a** | **0.034** | **21.539** | **0.000** | **0.494** |
| **A1b** | **A1d** | **B** | **0.021** | **11.290** | **0.000** | **0.321** |
| A1b | A2 | B | 0.002 | 0.836 | 0.202 | 0.032 |
| **A1c** | **A1d** | **A1a** | **0.009** | **4.889** | **0.000** | **0.234** |
| **A1c** | **A1d** | **A1b** | **0.010** | **7.128** | **0.000** | **0.341** |
| **A1c** | **A1d** | **A2** | **0.009** | **4.354** | **0.000** | **0.068** |
| **A1c** | **A1d** | **B** | **0.008** | **4.157** | **0.000** | **0.148** |
| **A2** | **A1c** | **B** | **0.010** | **3.771** | **0.000** | **0.178** |
| **A2** | **A1d** | **B** | **0.017** | **6.595** | **0.000** | **0.274** |

**Table S6**: Parameters of the best-fit speciation models. Parameters have been converted using estimated mutation rates[12] (μ_min_ = 6.09E-10 < μ_avg_ = 5.55E-10 < μ_max_ = 5.05E-10; values are given for the averaged mutation rate (μ_avg_), respective minima and maxima are in bracket). Unit for time is in kiloannum (ka).

|  | Best fit model | Nref | nu1a | nu2a | nu1b | nu2b | m | m12 | m21 | T1 | T2 | T3 |
| --- | --- | --- | --- | --- | --- | --- | --- | --- | --- | --- | --- | --- |
| A1/B | sec_contact_asym_mig_three_epoch | 221,088.8 (201,484.8 – 242,978.7) | 758,223.9 (690,992.3 – 833,295.6) | 434,386.5 (395,869.5 – 477,395.1) |  |  |  | 1.57E-05 (1.43E-05 - 1.73E-05) | 4.39E-06 (4E-06 - 4.82E-06) | 137.9 (125.7 – 151.6 | 0.112 (0.102 – 0.123) | 0.011 (0.010 – 0.012) |
| A1/A2 | sec_contact_asym_mig | 464,934.9 (423,709.1 – 510,968.1) | 178,535 (162,704.3 – 196,211.7) | 76,234.45 (69,474.74 – 83,782.41) |  |  |  | 1.05E-05 (9.53E-06 - 1.15E-05) | 3.68E-05 (3.35E-05 - 4.04E-05) | 101.8 (92.7 - 111.8) | 0.053 (0.049 - 0.058) |  |
| A2/B | sec_contact_sym_mig_size_three_ epoch | 157,540 (143,570.9 – 173,138) | 157,240.7 (143,298.1 – 172,809.1) | 169,159.5 (154,160.1 – 185,908) | 771,457.6 (703,052.5 – 847,839.5) | 30,221.66 (27,541.9 – 33,213.9) | 1.87E-05 (1.71E-05 - 2.06E-05) |  |  | 97.5 (88.9 - 107.2) | 0.081 (0.074 - 0.089) | 0.006 (0.005 - 0.007) |

m: symmetrical migration rate between species,

m12: asymmetrical migration rate from species 1 to species 2,

m21: asymmetrical migration rate from species 2 to species 1,

Nref: effective population size of ancestral species

nu1: effective population size of extant species 1, nu1a: effective population size of extant species 1 between speciation and the beginning of the second phase,

nu1b: effective population size of extant species 1 during the second phase,

nu2: effective population size of extant species 2,

nu2a: effective population size of extant species 1 between speciation and the beginning of the second phase,

nu2b: effective population size of extant species 1 during the second phase,

T and T1: time of speciation,

T2: duration of the second phase in two epoch models,

T3: duration of the third phase in three epoch models.

**Table S7**: Relaxed molecular clock-based estimates for species divergence time between the three species in relation with figure 4a.

|  | Divergence | Divergence time (ka) | Time interval  (95% HPD) |
| --- | --- | --- | --- |
| Node 1 | Calibration | 290 | 328.347 - 271.69 |
| Node 2 | A - B | 152.26 | 174.408 - 132.923 |
| Node 4 | A1 - A2 | 102.611 | 119.16 - 88.36 |
| Node 3 | B | 114.876 | 141.337 - 93.369 |
| Node 5 | A1a -A1bcd | 86.545 | 102.578 - 73.018 |
| Node 6 | A2 | 81.422 | 99.071 - 66.916 |
| Node 7 | A1b - A1cd | 72.327 | 87.614 - 59.708 |
| Node 8 | A1a | 68.39 | 85.251 - 54.863 |
| Node 9 | A1c - A1d | 59.244 | 73.747 - 47.592 |
| Node 10 | A1b | 56.878 | 72.347 - 44.717 |
| Node 11 | A1c | 46.431 | 60.488 - 35.641 |
| Node 12 | A1d | 45.883 | 60.249 - 34.253 |

**SI References**

1. Green JC, Course PA, Tarran GA. The life-cycle of *Emiliania huxleyi*: A brief review and a study of relative ploidy levels analysed by flow cytometry. *J Mar Sys* 1996; **9**: 33–44.

2. Bendif EM, Probert I, Young JR, von Dassow P. Morphological and phylogenetic characterization of new *Gephyrocapsa* isolates suggests introgressive hybridization in the *Emiliania/Gephyrocapsa* complex (Haptophyta). *Protist* 2015; **166**: 323–336.

3. Read BA, Kegel J, Klute MJ, Kuo A, Lefebvre SC, Maumus F, et al. Pan genome of the phytoplankton *Emiliania* underpins its global distribution. *Nature* 2013; **499**: 209–213.

4. Danecek P, Bonfield JK, Liddle J, Marshall J, Ohan V, Pollard MO, et al. Twelve years of SAMtools and BCFtools. *Gigascience* 2021; **10**: giab008.

5. Stamatakis A. RAxML version 8: a tool for phylogenetic analysis and post-analysis of large phylogenies. *Bioinformatics* 2014; **30**: 1312–1313.

6. Mirarab S, Reaz R, Bayzid MS, Zimmermann T, S. Swenson M, Warnow T. ASTRAL: Genome-scale coalescent-based species tree estimation. *Bioinformatics* 2014; **30**: i541–i548.

7. Darriba D, Posada D, Kozlov AM, Stamatakis A, Morel B, Flouri T. ModelTest-NG: A New and Scalable Tool for the Selection of DNA and Protein Evolutionary Models. *Mol Biol Evol* 2020; **37**: 291–294.

8. Rambaut A, Drummond AJ, Xie D, Baele G, Suchard MA. Posterior Summarization in Bayesian Phylogenetics Using Tracer 1.7. *Syst Biol* 2018; **67**: 901–904.

9. Bendif EM, Nevado B, Wong ELY, Hagino K, Probert I, Young JR, et al. Repeated species radiations in the recent evolution of the key marine phytoplankton lineage *Gephyrocapsa*. *Nat Commun* 2019; **10**: 1–9.

10. Malinsky M, Matschiner M, Svardal H. Dsuite - Fast D-statistics and related admixture evidence from VCF files. *Mol Ecol Resour* 2021; **21**: 584–595.

11. Filatov DA, Bendif EM, Archontikis OA, Hagino K, Rickaby REM. The mode of speciation during a recent radiation in open-ocean phytoplankton. *Curr Biol* 2021; **31**: 5439-5449.e5.

12. Krasovec M, Rickaby REM, Filatov DA. Evolution of Mutation Rate in Astronomically Large Phytoplankton Populations. *Genome Biol Evol* 2020; **12**: 1051–1059.

13. Forester BR, Lasky JR, Wagner HH, Urban DL. Comparing methods for detecting multilocus adaptation with multivariate genotype–environment associations. *Mol Ecol* 2018; **27**: 2215–2233.

14. Oksanen J, Blanchet G, Friendly M, Kindt R, Legendre P, McGlinn D, et al. vegan: Community Ecology Package. R package version 2.5-7. 2020.

15. Kruskal WH, Wallis WA. Use of ranks in one-criterion variance analysis. *J Am Stat Assoc* 1952; **47**: 583–621.

16. Portik DM, Leaché AD, Rivera D, Barej MF, Burger M, Hirschfeld M, et al. Evaluating mechanisms of diversification in a Guineo-Congolian tropical forest frog using demographic model selection. *Mol Ecol* 2017; **26**: 5245–5263.

17. Iassonova DR, Hammond EG, Beattie SE. Oxidative stability of polyunsaturated triacylglycerols encapsulated in oleaginous yeast. *J Am Oil Chem Soc* 2008; **85**: 711–716.

18. Young JR, Westbroek P. Genotypic variation in the coccolithophorid species *Emiliania huxleyi*. *Mar Micropaleontol* 1991; **18**: 5–23.

19. Hagino K, Bendif EM, Young JR, Kogame K, Probert I, Takano Y, et al. New evidence for morphological and genetic variation in the cosmopolitan coccolithophore *Emiliania huxleyi* (prymnesiophyceae) from the cox1b-atp4 genes. *J Phycol* 2011; **47**: 1164–1176.
